# Top-down control of planktonic ciliates by microcrustacean predators is stronger in lakes than in the ocean

**DOI:** 10.1038/s41598-022-14301-y

**Published:** 2022-06-22

**Authors:** Xiaoteng Lu, Thomas Weisse

**Affiliations:** 1 Research Department for Limnology, University of Innsbruck, Mondseestr. 9, 5310 Mondsee, Austria; 2 Present Address: Department of Biology, Shenzhen MSU-BIT University, 1 International University Park Road, Shenzhen, Guangdong Province P.R. China

**Keywords:** Community ecology, Ecosystem ecology, Freshwater ecology, Population dynamics, Biooceanography, Freshwater ecology, Population dynamics, Food webs, Ecology

## Abstract

Planktonic ciliates are major components of pelagic food webs in both marine and freshwaters. Their population dynamics are controlled ‘bottom-up’ by prey availability and ‘top-down’ by microcrustacean predators. In oceans, copepods are the main ciliate predators while in lakes cladocerans are the typical predators. The efficacy by which these functionally different predators control ciliate population dynamics is debated. We, therefore, investigated experimentally the grazing of three microcrustacean predators with different feeding modes on five freshwater ciliates. We then performed a meta-analysis to assess if our findings can be generalised for aquatic ecosystems. We hypothesized that top-down control is stronger in lakes than in the ocean. We find that: (*i*) average ingestion rates of marine and freshwater microcrustaceans do not differ; (*ii*) clearance rates of freshwater cladocerans decrease with ciliate size but increase with ciliate size in freshwater copepods; (*iii*) clearance rates of the marine microcrustaceans is unrelated to ciliate cell size. These findings have implications for the functioning of freshwater and marine food webs: (*i*) the ciliate—microcrustacean link is stronger in lakes than in the ocean, and (*ii*) globally top-down control of ciliates is unlikely in the ocean.

## Introduction

The ciliated protozoa (ciliates, phylum Ciliophora Doflein, 1901) are major players in most pelagic food webs on Earth^1,2^. Ciliates sit at the interface between the microbial and classical pelagic food web and are central to transferring phytoplankton production to higher trophic levels. Although they are a phylogenetically homogenous group, ciliates are morphologically and physiologically diverse, representing ~ 8000 species^3^. Therefore, in terms of ecological importance and taxonomic diversity, ciliates are ideal models of pelagic eukaryotic microbial functional diversity, across fresh and marine water.

Top-down control by different functional groups of microcrustacean predators (cladocerans and copepods, mostly < 2 mm in body length) is crucial for shaping the ciliate communities, both in marine and freshwater^4–6^. Most planktonic ciliates fall within the prey size spectrum of microcrustaceans^7–9^. Consequently, predation on ciliates by different microcrustaceans has been studied both in the laboratory and in situ experiments (compiled in the Dataset, see Data availability).

Cladocerans, calanoid, and cyclopoid copepods have overlapping food spectra and co-occur in most lakes, and the two copepod orders are also common in the ocean. Functionally, these three microcrustacean taxa represent different guilds. Daphnids are efficient filter feeders (feeding-current feeders *sensu*^10^), ingesting suspended particles ranging in size from bacteria to algae, ciliates, and small rotifers but preferring food < 30 µm^11–13^. Calanoid copepods such as the freshwater genus *Eudiaptomus* and the similar marine genus *Acartia* are mixed-type feeders, feeding more selectively by actively grasping larger particles (ambush feeding^10^) and filtering small particles^11,14^. Although calanoids may feed on smaller particles, their feeding becomes efficient only at prey size of ~ 10 µm^15,16^. Cyclopoid copepods are exclusively ambush feeding species that actively hunt their relatively large, mostly motile prey^10,11^.

In freshwater lakes, microcrustaceans are species-rich (e.g., with > 600 species of cladocerans^17^) and are the primary ciliate predators. Several species of *Daphnia* reach high abundances (> 50 individuals L^−1^) in many lakes and, due to their high filtration efficiency, can exert strong top-down control on the microbial food web through predation, mechanical interference or both^8,18,19^. In the ocean, ciliate predators are more diverse and less abundant. Copepods play a major role in ciliate predation^20,21^, but other plankton organisms such as mucous net feeders (mainly pelagic tunicates and pteropods) and ctenophores and cnidarian medusae that use an entangling feeding strategy^22^ may also feed upon ciliates in the ocean^4,11,23^. Cladocerans are quantitatively negligible in the open sea; the eight cosmopolitan marine cladocerans are primarily coastal species^24,25^.

Controversial evidence exists on the efficacy by which the different predator types control ciliate population dynamics in lakes^12,26,27^. Due to their contrasting feeding behaviour, size preferences and abundances, we hypothesised that the top-down effects of daphnids on planktonic ciliates should be stronger than that of copepods (first hypothesis, H_1_). Specifically, we expected that daphnids would prefer the more numerous, smaller ciliates, while cyclopoid copepods would prefer the less abundant, larger ciliates (H_2_).

Mainly due to the absence of *Daphnia* and similar cladocerans in the ocean, the taxonomic and, by implication, the functional role of microcrustaceans should differ between freshwater and marine ecosystems. On a global average, the net primary production of lakes (260 g C m^−2^ y^−1^) is almost twice as high as that of the ocean (140 g C m^−2^ y^−1^)^28,29^, suggesting that top-down control of autotrophs and primary consumers may be stronger in lakes than in the ocean (H_3_), where bottom-up control via resource limitation generally prevails (reviewed by More et al*.*^30^).

To test the above hypotheses, we first used laboratory experiments to investigate top-down control by three functionally different microcrustacean predators; i.e. a daphnid (*Daphnia* sp.), a calanoid copepod (*Eudiaptomus* sp.), and a cyclopoid copepod (*Cyclops* sp.) on five planktonic freshwater ciliates: *Urotricha* sp., *Vorticella natans*, *Histiobalantium bodamicum*, *Strobilidium caudatum* and *Rimostrombidium lacustris*. These ciliates represent typical species in mesotrophic lakes and differ in size and swimming behaviour. Four of the five species swim fast or intermittently and can 'jump' (Table 1), which is an effective strategy to escape microcrustacean predation^31,32^. We measured the ingestion and clearance rates of each microcrustacean predator in relation to ciliate species and size. Secondly, we performed a meta-analysis to detect if our findings can be generalised and to test for functional differences between top-down control of ciliates in lakes and the ocean. Finally, since our statistical analyses supported H_3_, we discuss the important implications of our findings for the functioning of freshwater and marine food webs.

**Table 1:** Average cell size, volume, carbon biomass and swimming behaviour of the study ciliates.

Ciliate species	Cell length (µm)	Cell width (µm)	Cell volume (µm^3^)	Cellular biomass (ng C cell^−1^)	Swimming speed	Jumps
*Urotricha* sp.	18	14	1452	0.14	Fast	Yes
*Vorticella natans*	41	36	28,250	3.27	Slow	No
*Histiobalantium bodamicum*	53	38	25,929	3.01	Intermittent	Yes
*Strobilidium caudatum*	59	47	67,939	7.44	Fast	Yes
*Rimostrombidium lacustris*	73	66	166,772	17.30	Fast	Yes

Biomass was converted from volume assuming pg C cell^−1^ = 0.261 × volume^0.860^ if cell volume is less than 3000 µm^3^ and pg C cell^−1^ = 0.216 × volume^0.939^ if cell volume exceeds 3000 µm^3^^68^.

## Results

### Experimental results—microcrustacean ingestion rates

Ingestion rates of each microcrustacean were significantly affected by ciliate species (*p* < 0.001) but appeared unaffected by predator species (*p* = 0.305, Suppl. Table S1).

**Figure 1:**
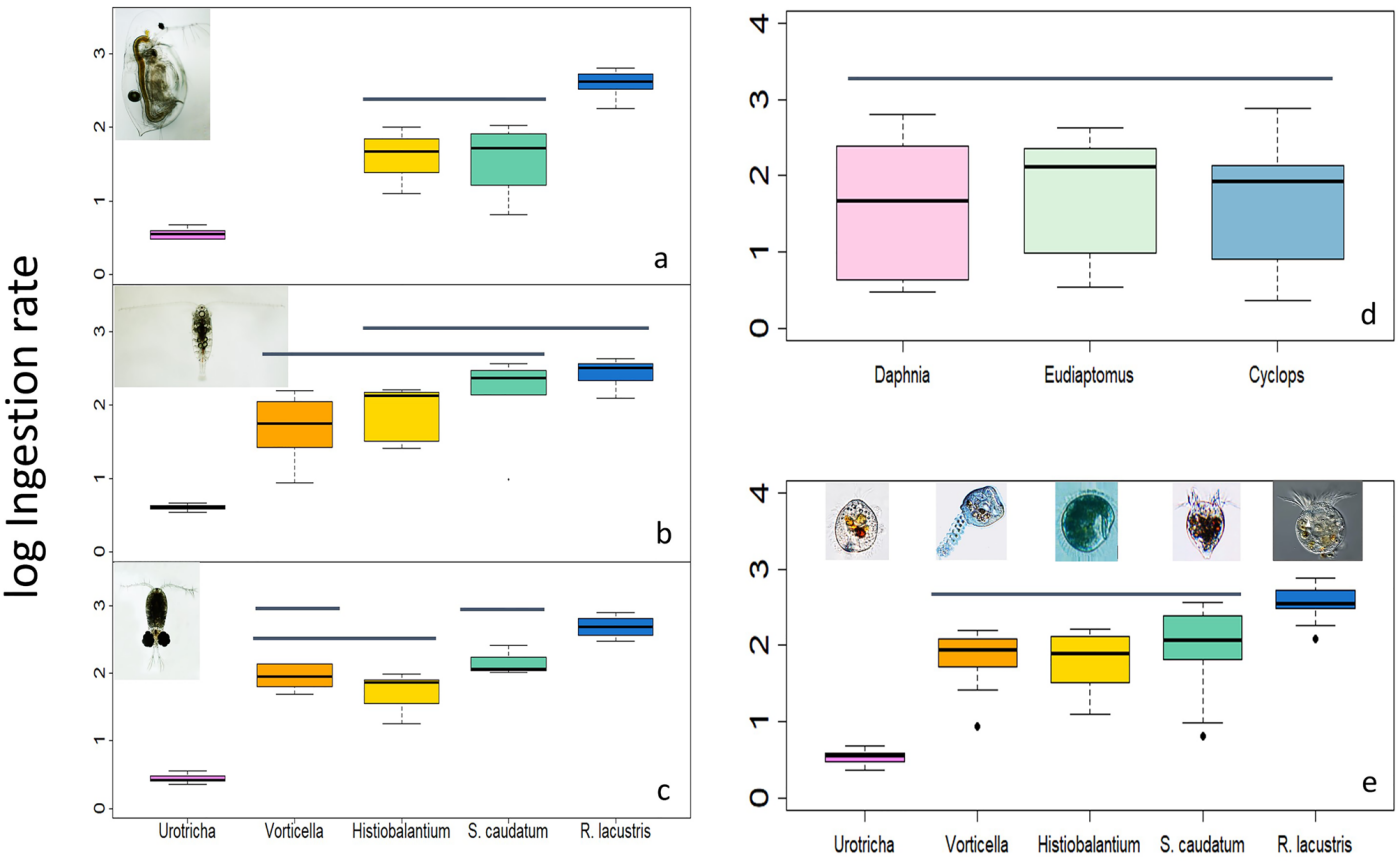
Box plots of log_10_–transformed ingestion rates (logIR, ng C ind^−1^ d^−1^) of the three microcrustacean predators. (**a**–**c**) LogIR of *Daphnia* (**a**), *Eudiaptomus* (**b**), and *Cyclops* (**c**) on the five ciliate species; (**d**) combined logIR of the three microcrustacean species; (**e**) combined logIR of all predators on each ciliate species. The boundary of the box closest to zero indicates the 25th percentile, the solid line within the box marks the median, and the boundary of the box farthest from zero indicates the 75th percentile. Error bars above and below the box indicate the 90th and 10th percentiles. The horizontal bars indicate logIR that were not significantly different.

Ciliate-specific ingestion rates by all predators were only in a few cases significantly different (Fig. 1a–c,e). In all cases, ingestion was lowest on *Urotricha* sp. and highest on *Rimostrombidium lacustris*. The ingestion rates of the three predators on all ciliates combined did not differ (Fig. 1d), and the mean microcrustaceans ingestion rate averaged over all treatments was 144 ± 174 ng C individual^−1^ d^−1^. The mean ciliate-specific ingestion rates of the microcrustaceans ranged from 3 ± 7 ng C individual^−1^ d^−1^ measured for the smallest ciliate (*Urotricha* sp.) to 398 ± 173 ng C individual^−1^ d^−1^ for the largest ciliate (*R. lacustris*) (Fig. 1e).

Log_10_–transformed ingestion rates were positively related to ciliate cell size for all predators (Fig. 1e, Suppl. Fig. S1). In all cases, ordinary least squares regression (OLS) yielded the best model fit and significant parameter estimates (Suppl. Table S2). The OLS models explained between 76 and 88% of the total variance (R^2^ in Table S2).

Removing one outlier each for clearance and ingestion rates did not affect the conclusions in any of the models tested.

### Experimental results—microcrustacean clearance rates

**Figure 2:**
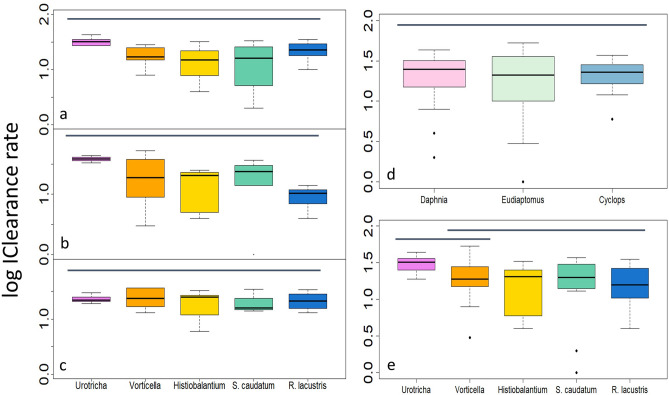
Log_10_–transformed clearance rates (logCL, mL individual^−1^ d^−1^) of *Daphnia* (**a**), *Eudiaptomus* (**b**) and *Cyclops* (**c**) on the five ciliate species and on all ciliates combined (**d**). Combined logCL of the predators on each ciliate species is shown in (**e**). The horizontal bars indicate logCL that were not significantly different from each other.

Clearance rates (*CL*) of all three predators did not depend on ciliate species (Fig. 2a–c and Suppl. Table S1) and the average *CL* of the individual predators did not differ (Fig. 2d). Averaged over all treatments, *CL* was 22 ± 12 mL individual^−1^ d^−1^. Mean ciliate-specific *CL* varied 1.7-fold, from 18 ± 10 mL individual^−1^ d^−1^ measured for *R. lacustris* to 31 ± 8 mL individual^−1^ d^−1^ for *Urotricha* sp. (Fig. 2e).

**Figure 3:**
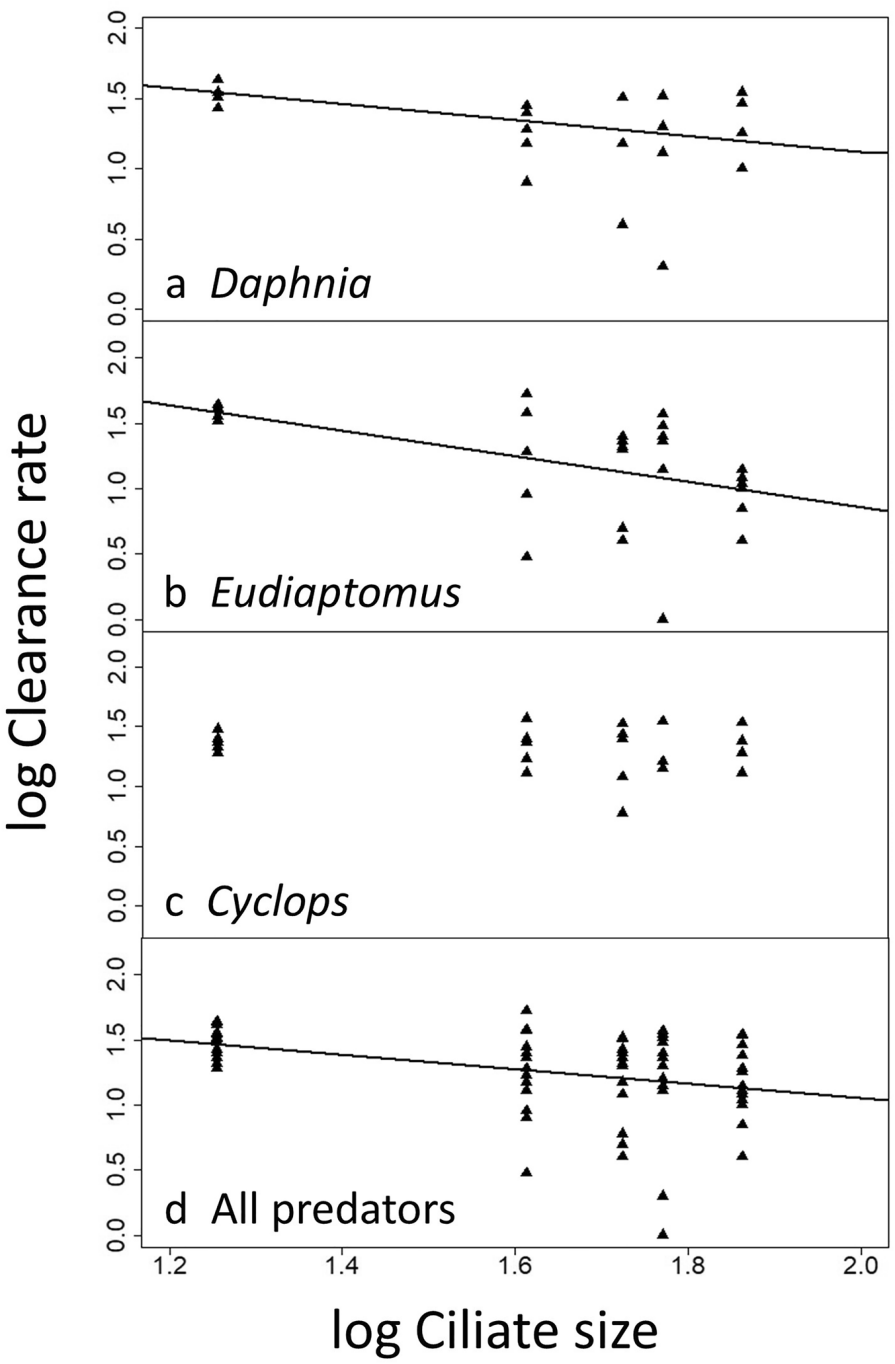
Log_10_–transformed clearance rates (logCL, in mL individual^−1^ d^−1^) of the three predators (a–c) and all predators combined (d) vs ciliate size. Data were fitted by a power curve (y = ax^b^; plot a) and by OLS (y = kx + c; plots b, d).

In *Cyclops*, log-transformed *CL* were unrelated to ciliate size (Fig. 3c). In *Daphnia*, *CL* declined with ciliate size (Fig. 3a); power curve fitting yielded significant estimates for both parameters characterising the model (Suppl. Table S2). In *Eudiaptomus* (Fig. 3b) and for all predators combined (Fig. 3d), *CL* were also inversely related to ciliate size, supported by all three curve fitting functions with similar AIC scores (Suppl. Table S2). However, compared to ingestion rates, the variation of logCL explained by ciliate cell size was smaller (< 30%, Table S2).

### Meta-analysis—grazing rates of ciliates by marine and freshwater microcrustaceans

**Figure 4:**
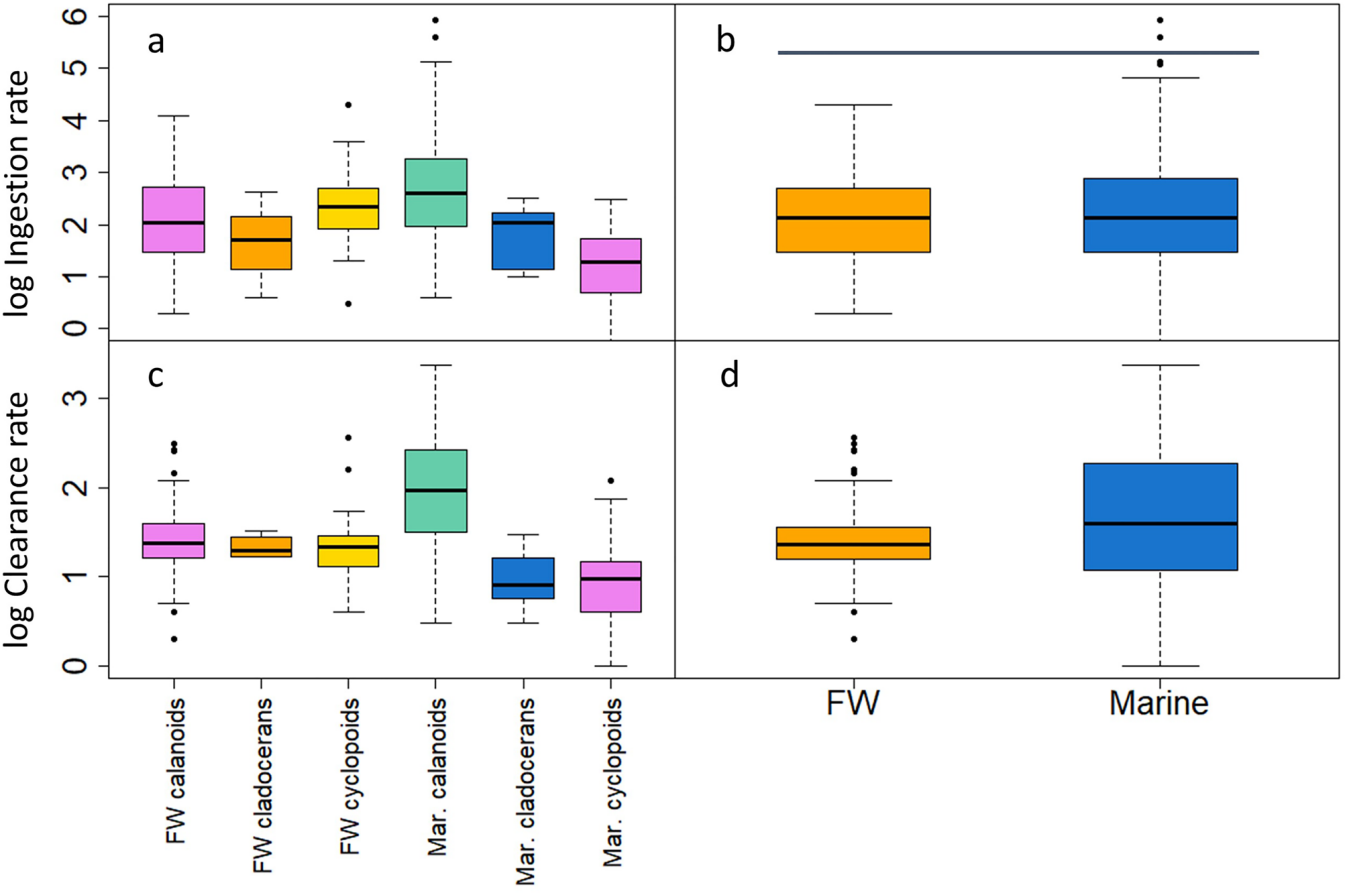
Log_10_–transformed ingestion rates (logIR, in ng C ind^−1^ d^−1^, **a**,**b**) and clearance rates (logCL, in mL individual^−1^ d^−1^, **c**,**d**) of major functional groups of microcrustaceans in freshwater (FW) and marine waters. In (**b**) and (**d**), all predators were combined for the two habitats. The horizontal bar in (**b**) indicates logIR of freshwater and marine predators were not significantly different from each other. Significant differences between individual functional groups (**a**,**c**) are reported in the text and Suppl. Table S4.

More than 50 species of microcrustaceans have been reported to impose moderate to high grazing rates on various planktonic ciliates (see Dataset in Supplementary Information). We differentiated six functional predator groups, i.e. freshwater cladocerans, freshwater calanoids, freshwater cyclopoids, marine cladocerans, marine calanoids and marine cyclopoids in the following analyses. The mean clearance rate of freshwater microcrustacean predators (37 ± 61 mL individual^−1^ d^−1^, Fig. 4d) is higher than our experimental results reported above (22 ± 12 mL ind.^−1^ d^−1^) and more than fivefold lower than that of their marine counterparts (211 ± 383 mL ind.^−1^ d^−1^). The large calanoid species *Epischura lacustris* and the smaller species *Boeckella hamata* known from New Zealand lakes^27,33^ strongly affected the mean values of freshwater calanoids and all freshwater microcrustaceans together; without these two species, the latter is reduced to 24 ± 36 mL individual^−1^ d^−1^), i.e. not significantly different from our experimental results. The high clearance rate of marine microcrustaceans (Fig. 4d) is mainly caused by large calanoid copepod species of the genera *Calanus* and *Neocalanus*.

In addition to the difference in the clearance rate between marine and freshwater predators reported above, the results from our LM analyses (Suppl. Table S3), one-way ANOVA, and Tukey HSD tests indicate that: (*i*) clearance rates differ significantly between several functional groups (Suppl. Table S4) and marine calanoid copepods have the highest average clearance rate (Fig. 4c); (*ii*) however, the clearance rate of FW cyclopoids is not different from that of freshwater calanoids, FW cladocerans, marine cyclopoids and marine cladocerans; (*iii*) ingestion rates of freshwater calanoids, freshwater cladocerans, freshwater cyclopoids and marine cladocerans do not differ (Fig. 4b and Suppl. Table S4) and, in contrast to *CL*, (*iv*) the habitat has no effect (Suppl. Table S3), i.e. there is no difference between the ingestion rates of freshwater and marine predators on ciliates (Fig. 4b).

**Figure 5:**
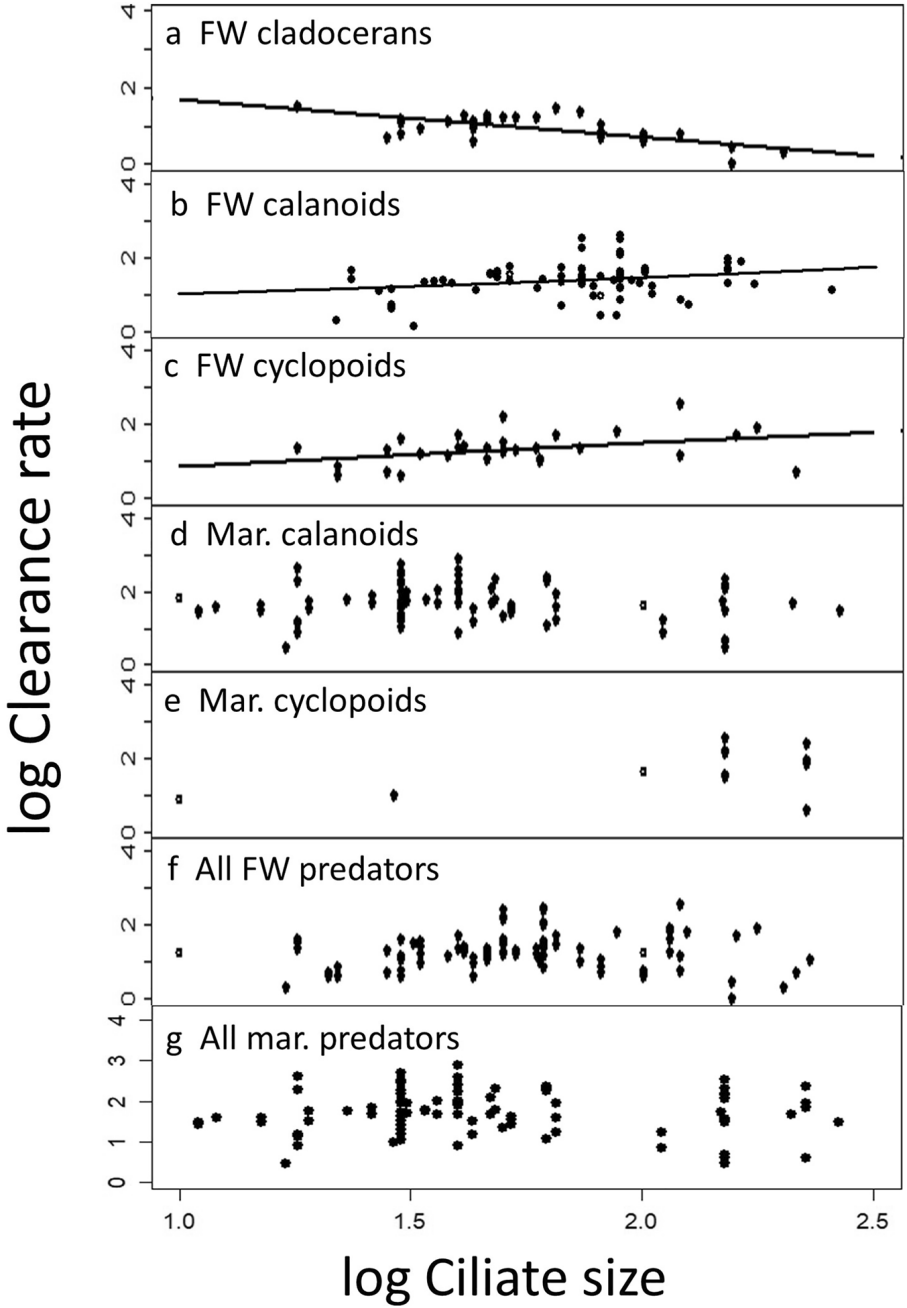
Log_10_–transformed clearance rates (logCL, in mL individual^−1^ d^−1^) of the major functional groups of microcrustacean predators vs the ciliate size. Data were fitted by linear regression (a) and a power function (b,c), respectively. Curve fitting failed for the data shown in panels d–g. FW denotes freshwater, Mar and mar = marine. All model results are reported in Suppl. Table S5.

Our analysis of size-specific ciliate clearance rates (Fig. 5; Suppl. Table S5) yielded that: (*i*) clearance rates decline with ciliate size in freshwater cladocerans (Fig. 5a) and (*ii*) increase with ciliate size for freshwater calanoids (Fig. 5b) and freshwater cyclopoids (Fig. 5c); (*iii*) for marine calanoids (Fig. 5d), marine cyclopoids (Fig. 5e), and all freshwater (Fig. 5f) and all marine predators (Fig. 5g) combined, either one or both parameters characterising the respective curves were not significant (Suppl. Table S5), i.e. the available data do not allow to draw any clear conclusions on the size preference of these groups.

## Discussion

### Conceptual difference between ingestion rates and clearance rates

We reported **i**ngestion rates and clearance rates in this study because they characterise different aspects of the feeding behaviour of microcrustaceans. From a predator's point of view, the ingestion rate, which is a direct measure of food uptake, is more relevant^11^. At comparable prey levels, the amount of food ingested increases with prey size, as we have demonstrated for freshwater microcrustaceans feeding on ciliates (Suppl. Fig. S1). Due to the paucity of data, we could not test if this also applies to marine copepods. Clearance rate, i.e. the volume of water cleared of food particles by a predator per unit time, is more important for the prey than ingestion rates^11^. The community clearance rate (or community grazing rate, G^34^) of all predators in a population provides an estimate of the grazing effect on a prey population. If G exceeds the growth rates of ciliates or other prey, their population sizes will be reduced. Since our aim was to analyse top-down control of planktonic ciliates, we focus the following discussion on clearance rates.

### Maximum clearance rates of filter feeders and ambushers feeding on planktonic ciliates may be similar—experimental evidence and theoretical background

The three microcrustacean species that we studied represent feeding-current feeding (*Daphnia*), ambush feeding (*Cyclops*), and a combination of both, i.e. mixed-type feeding (*Eudiaptomus*). We found in our experimental study that (*i*) clearance rates of *Daphnia*, *Eudiaptomus* and *Cyclops* fed with five planktonic ciliate species do not differ (Fig. 2d) and that (*ii*) this result can be partially generalised for all available data obtained from our literature search: *CL* of freshwater cyclopoids do not differ from those of freshwater calanoids and freshwater cladocerans (Suppl. Table S4). However, in contrast to our assumption stated in the introduction, our meta-analysis demonstrated that *CL* of freshwater cladocerans are not higher but significantly lower than those of freshwater calanoids (Fig. 4c, Suppl. Table S4). Therefore, we reject our first hypothesis (H_1_).

Similar experimental results were recently reported for marine copepods, i.e. the small cyclopoid *Oithona nana* (ambush feeding) and the calanoids *Temora longicornis* (feeding-current feeding) and *Centropages hamatus* (cruising-feeding)^35^. Almeda and colleagues^35^ reported that the maximum clearance rate of the ambush feeder did not differ when feeding on motile prey ranging in size from 7 to 40 µm.

The feeding of planktonic predators can be analysed by their functional response (FR), which describes their ingestion and clearance rates as functions of the prey levels^36^. Maximum clearance rate (*CL*_*max*_) is a useful parameter to compare the feeding performance across different taxa^37,38^. If not derived from FR equations^35,39^, *CL*_*max*_ can be obtained at low prey levels; this study) because the clearance rate usually decreases with increasing prey concentrations (Holling's type-I and type-II FR).

Since the encounter rate between predator and prey is higher in active feeders than in ambushers that prefer a “sit-and-wait” foraging strategy^35^, the latter feeding behaviour leads to lower clearance rates than the former for non-motile prey such as diatoms^10,35^. Active ambush-feeding copepods search for prey by perceiving the hydrodynamic disturbance that the prey generates^10^. Because clearance rates vary with prey detection distance squared, a small increase in prey detection distance may compensate for the reduced encounter rates, relative to actively feeding copepods^35^. As Almeda and co-workers^35^ point out, this is the plausible explanation why *Oithona* reached similar *CL*_*max*_ as *Temora* and *Centropages* for motile prey in their study, which was supported for freshwater cyclopoids and calanoids by the results from the experiments and the meta-analysis of the present study.

### Top-down control of planktonic ciliate populations in the ocean and lakes

The extent to which planktonic ciliate populations are controlled top-down depends mainly on the balance between ciliate growth rates and their grazing loss rates imposed by predators. Other losses due to senescence and parasitism^40,41^ may be important at times but appear to be negligible on a global scale.

Maximum clearance rates of marine copepods ranging from 0.11 to > 3000 mL ind.^−1^ d^−1^ have been reported (see Data availability), and carbon-specific clearance rates vary more than tenfold^42^. In contrast to the present work, the studies reviewed by Brun and co-workers^55^ comprised virtually all copepod instars and considered different food sources. The average maximum clearance rate of 25 marine copepod species feeding on mixed diets, including many diatom species, was 183 ± 719 mL ind.^−1^ d^−1^^42^, i.e. somewhat lower than our ciliate-specific estimate (211 ± 383 mL ind.^−1^ d^−1^). Although due to the large standard deviations (this difference is not significant) it may support the assumption that ambush feeding copepods prefer the motile ciliates and dinoflagellates over non-motile phytoplankton^23,43^.

In contrast to a previous review on ciliate–copepod interactions^20^, reliable global estimates of marine copepod abundance are now available^44^. However, to our knowledge, this is not the case for freshwater copepod abundance and ciliate growth rates in the epipelagic zone of the ocean and the epilimnion of lakes. The global median epipelagic (0–200 m) biomass of mesozooplankton (0.2–2.0 mm) is 2.7 µg C L^−1^^44^. Assuming copepods account for 80% of the abundance and biomass in this size class^45,46^ and average *per capita* biomass of 7.3–10 µgC individual^−1^^20,45^, we obtain a global average copepod abundance of 0.2–0.3 individuals L^−1^ in the upper 200 m of the ocean. The abundance of omnivorous copepods in the central gyres of the ocean is even lower, < 0.1 individuals L^−1^^47^. Further assuming the average clearance rate that Brun and co-workers^55^ obtained for marine microcrustaceans, Eq. (2) predicts that copepods would collectively graze ciliates at a rate of 0.018–0.055 d^−1^. This estimate of G (community grazing rate, see “Conceptual difference between ingestion rates and clearance rates” section) yields the ciliate growth rate needed to maintain their standing stock.

Experimental evidence for low copepod grazing on marine ciliates was provided by Armengol and co-workers^43^ for natural microplankton communities in an oligotrophic area of the NE Atlantic at comparable prey and predator levels to those used in the present study. At copepod densities ≤ 10 copepods L^−1^, their cumulative grazing rates on ciliates ranged from 0.01 to 0.03 d^−1^, which is close to our above estimate.

Specific maximum growth rates (*µ*_*max*_) of 77 ciliate species or strains from marine and freshwater measured in the laboratory under prey-saturating conditions range from < 0.01 to 6.8 d^−1^, with a mean value of 1.1 d^−1^ (reviewed by^48^). However, *µ*_*max*_ is rarely reached under food-limited conditions in the natural realm^8,38^. Studies on different marine ciliates showed that their mean population growth rates in the absence of predators is close to 0.6 d^−1^^49,50^, but these estimates are biased for tintinnids and may not represent typical oceanic conditions. Yet, even if ciliate in situ growth rates in the ocean are tenfold lower than their respective *µ*_*max*_, ciliates would still outgrow predation losses by copepod grazing.

Irrespective of the uncertainties inherent in such global estimates, both the review by Calbet and Saiz^20^ and the present study strongly suggest that top-down control of ciliates by copepods is highly unlikely in the ocean, as conjectured earlier^16^. This conclusion was supported by Armengol and colleagues^43^ and similar experimental studies with natural plankton reporting that copepods would typically remove 2–5% d^−1^ of the ciliate standing stock in the ocean^51,52^. This does not preclude that at times when copepods occur in swarms, they may keep ciliate growth in check or even reduce ciliate numbers^20,53^, but those are local phenomena in the oligotrophic ocean. In more productive areas such as upwelling regions and coastal waters, copepods may have a much stronger effect on ciliate populations, and bottom-up control by food resources and temperature and top-down control by copepods may alternate seasonally^20,51,54^.

Although copepod instars are usually more numerous than adult copepods^55^, the ingestion and clearance rates of nauplii and early copepodites are much lower than those of adults^21,35,42,56^. Furthermore, feeding rates of calanoid nauplii at low food levels in the oligotrophic ocean (< 100 µg C L^−1^) are approximately one order of magnitude lower (< 0.2 µg C individual^−1^ d^−1^) than maximum rates obtained in the laboratory, indicating strong food limitation^21^. The abundance of other ciliate metazoan grazers in the central gyres seems to be too low to control ciliate populations efficiently^57^ but the role of carnivorous protozooplankton and intraguild predation by other ciliates and dinoflagellates^23,58^ awaits further research. By implication, ciliates in the warm oligotrophic ocean should primarily be controlled by resources, i.e. by their nanosized food (small algae and heterotrophic protists^59^). Positive correlations between phytoplankton and ciliate biomass observed on the global average^20^ and in specific regions^51^ support this conclusion.

Does the lack of top-down control of ciliates by microcrustaceans also apply to lakes? In the present study, the mean growth rate of all five freshwater ciliate species at moderate food supply was 0.32 d^−1^. Assuming the mean clearance rate that we measured (22 mL individual^−1^ d^−1^), it would require ~ 15 microcrustaceans L^−1^ to fully control ciliate dynamics. Ciliates are suppressed by *Daphnia* whenever the latter reach high abundances (>> 10 ind L^−1^), which typically occurs in mesoeutrophic temperate lakes during spring and early summer^60^. In Lake Constance, which is probably the lake where ciliate–microcrustacean interactions have been studied in the most detail, the typical combined abundances of cladocerans, calanoid and cyclopoid copepods range from 15 to 30 ind L^−1^ during the summer half-year^61^ and higher abundances of microcrustaceans occur in many more eutrophic lakes and ponds (reviewed by Wetzel^62^). As an example, the omnivorous cyclopoid copepod *Mesocyclops thermocyclopoides* may reach up to 100 individuals L^−1^ in shallow, eutrophic subtropical and tropical water bodies and impose strong top-down control on the microbial food web including ciliates^63^.

In conclusion, although the evidence for temporary or even permanent control of ciliate communities by microcrustacean grazing mainly originates from temperate, mesotrophic and moderately eutrophic lakes, top-down control of ciliate comunities is more common in lakes than in the central ocean, supporting our third hypothesis (H_3_).

### Top-down effects of microcrustaceans in relation to ciliate size

Ciliates’ size influences their susceptibility to microcrustacean predators (see above, Introduction) but the evidence on the ‘ciliate size preference’ by different predators is equivocal^7,18,26,64,65^. We found an inverse relationship between ciliate size and clearance rates in *Daphnia* and *Eudiaptomus*, whereas the clearance rate of *Cyclops* showed no trend with ciliate size. The meta-analysis partially supported our results; i.e. clearance rates of freshwater cladocerans were inversely related to ciliate size, whereas clearance rates of freshwater cyclopoids and freshwater calanoids increased with ciliate size, as expected (H_2_). The fact that we did not find a positive relationship between ciliate size and clearance rate of *Cyclops* in our experimental study may have originated from the smaller size range of ciliates (~ 20–70 µm, Table 1) that we investigated compared to two previous studies that included ciliate species > 100 µm^39,63^. Accordingly, in lakes where *Daphnia* dominates the grazing pressure by microcrustaceans should primarily suppress the abundant, small and medium-sized ciliates (< 50 µm). In the oligotrophic ocean, where small omnivorous copepods prevail^43,47^, more experimental studies are needed to decipher if top-down effects of microcrustaceans are related to ciliate size^57^.

### Other factors potentially affecting ciliates' susceptibility to predation

As indicated by the large unexplained variance of the regression models (Suppl. Table S5), other factors than cell size have influenced the experimental results reported in this study and the literature. In particular, the swimming and jumping behaviour of ciliates is known to affect their susceptibility to microcrustacean predation^31–33^. In ciliates, swimming velocity is relatively invariant with cell size^37^. Although we did not measure the swimming velocity of our study ciliates, visual inspection suggests that the smallest species, *Urotricha* sp., swims and 'jumps' at similar speed to the larger species.

Surprisingly, we found no difference in the microcrustacean feeding rates on the slowly swimming species *Vorticella natans* and the similar-sized *Histiobalantium bodamicum*^66^, in contrast to the earlier conjecture that *V. natans* is more strongly top-down controlled by predation than *H. bodamicum*^67^. The latter species uses an intermittent swimming behaviour, with immotile floating periods interrupted by sudden, very fast jumps. Toxicysts, i.e. specialised extrusomes, which are another efficient defence mechanisms of some ciliates^32^, are not found in *V. natans*. Unpalatability due to biochemical composition has been demonstrated for at least one other peritrich ciliate, but little is known on chemical defences of planktonic ciliates^8^.

## Conclusions

This study provided general implications for the coupling between ciliates and their microcrustacean predators in freshwater and marine food webs. Our most important finding is that top-down control of ciliate populations by copepod grazing in the ocean is, on the global average, of minor quantitative importance. The low grazing pressure and the seeming lack of size-selective feeding by copepods on marine ciliates may cascade down to lower trophic levels, favouring picoplankton at the expense of nanoplankton in the nutrient-poor open sea. More experimental work on the ciliate–calanoid and ciliate–cyclopoid interactions in the oligotrophic ocean is needed to test these conclusions.

The ciliate–microcrustacean link is generally stronger in lakes, and ciliate populations may be reduced if the collective abundance of cladocerans and copepods is > 15 individuals L^−1^, irrespective if daphnids, calanoid, or cyclopoid copepods prevail.

We support an inverse relationship between ciliate size and clearance rates in daphnids and a positive relationship for freshwater cyclopoid copepods. Therefore, despite similar clearance rates, daphnids and cyclopoid copepods exert differential grazing pressure on the ciliate community in lakes. Since daphnids are more numerous than cyclopoid copepods in most natural lakes, top-down control by microcrustaceans is most efficient on small to medium-sized ciliates in lakes.

## Methods

### Study organisms

The microcrustaceans and ciliates species used in this study were isolated from Lake Mondsee, Austria (mesotrophic, 68 m deep, 47°49′41.88″N, 13°22′46.56″E). The phototrophic flagellate *Cryptomonas* sp. strain 26/80, which served as food for the ciliates and *Daphnia*, was obtained from the Algenkultursammlung Göttingen (SAG).

The predator species used in the experiments were female *Daphnia* and adult or late copepodite stages (C4–C6) of copepods. Most of the *Daphnia* specimens belonged to *Daphnia hyalina* (1.8 × 0.9 mm); however, some individuals were hybrids of *D. hyalina* and *D. galeata* (*Daphnia* × *obscura*). Adult cyclopoid copepods belonged to the subspecies *Cyclops abyssorum prealpinus* (1.2 × 0.4 mm). The calanoid copepods were identified as *Eudiaptomus gracilis* (1.1 × 0.3 mm). The taxonomic affiliation of some copepodites was not unequivocally clear; therefore, we refrain from reporting the results of the microcrustacean predation at the species level in the following.

The ciliate species used in the experiments were: the prostomatid *Urotricha* sp., the free-swimming peritrich *Vorticella natans*, the scuticociliate *Histiobalantium bodamicum*, and the choreotrich ciliates *Strobilidium caudatum* and *Rimostrombidium lacustris*. Cell volumes were calculated from lengths and widths measurements (Table 1) assuming appropriate geometric shapes and were converted to carbon biomass (ng C cell^−1^) using appropriate conversion factors (Table 1).

### Experimental design

The experiments were conducted under a 14:10 h Light: Dark photoperiod at 13–18 µmol photon m^−2^ s^−1^, which simulated the natural light cycle in spring through autumn at ~ 5–10 m depth in Lake Mondsee, Austria^69^. The experimental temperature was 15 °C, which is close to the mean temperature in the epilimnion of the lake between May and October^70,71^.

Experiments were conducted in transparent 100-mL cell culture flasks (CytoOne^®^). Each experiment consisted of two treatments (A, B) with six replicates each and lasted for 24 h. Treatment A (experimental flasks) contained one predator, one ciliate species and *Cryptomonas* sp. as food for the ciliates. Treatment B (controls) contained the same ciliate species and *Cryptomonas* sp. but no predators. Treatment A was used to calculate changes of ciliate abundance in the presence of predators, whereas treatment B was used to calculate changes of ciliate abundance in the absence of predators. Each experimental flask received one individual of the respective predator, i.e. the theoretical predator density was 10 individuals L^−1^ in the microcosm experiments.

Ciliates were added to each experimental container at a target concentration of 1 cell mL^−1^. The food organism, *Cryptomonas* sp., was added to reach an experimental abundance of 1.5 × 10^4^ mL^−1^, corresponding to ~ 0.5 mg C L^−1^^72^. The prey and predators were acclimatised to the experimental conditions for 24 h.

### Abundance and size estimates

*Cryptomonas* sp. cells were counted and sized alive by an electronic particle counter (CASY^®^ 1 Modell TTC). Ciliates were fixed with Lugol's solution and counted by inverted microscopy in counting chambers of 50–mL volume. The cell size of live ciliates was measured from images taken by an imaging cytometer (FlowCam^®^, Flow Cytometer And Microscope FlowCam, Fluid Imaging technology, Yarmouth, ME, USA)^69,73^ and analysed by an image analysis system (NIS elements D; Nikon CEE GmbH) connected to an inverted microscope.

*Daphnia* sp. was fixed with formalin-40% sucrose solution at the end of each experiment, and copepods were narcotised with filtered lake water amended with carbon dioxide before sizing. Lengths and widths were measured from the anterior margin of the head to the base of the posterior spine in *Daphnia* and from the anterior margin of the head to the posterior margin of the urosome in copepods (i.e. caudal rami were not included in measurements)^74^.

### Data acquisition and analysis

Microcrustacean grazing rates (*g*, d^−1^) were calculated according to Frost^75^:

1 $$g= \frac{\mathrm{ln}\left(\frac{{Cc}_{t}}{Cc0}\right) - \mathrm{ln}(\frac{{C}_{t}}{C0})}{\left({\mathrm{t}}_{1}-{\mathrm{t}}_{0}\right)}$$

where *Cc*_*0*_ and *Cc*_*t*_ are the initial (at t_0_) and final (at t_1_) ciliate numbers in the controls without predators and *C*_0_ and C_*t*_ are the initial and final ciliate concentrations in the containers with microcrustaceans.

Microcrustacean clearance rates (*CL*, mL individual^−1^ d^−1^) were obtained from grazing rates (*g*) and predator abundance (*P,* individual mL^−1^):

2 $$CL=\frac{g}{P}$$

Microcrustacean ingestion rates (*IR*, ng C individual^−1^ d^−1^) were calculated as

3 $$IR=CL \times m\times Rm$$

where *m* is the ciliate cellular biomass (ng C cell^−1^) and *Rm* (cells mL^−1^) is the geometric mean of ciliate abundance in the experimental containers with microcrustaceans.

Statistical analyses were conducted using the software R 4.0.5^76^, including the following packages: "stats", "lme4" and "effects" for the linear model and linear mixed model analyses; "car" for Analysis of Variance (ANOVA) procedures; "AICcmodavg" and "MuMIn" for model selection based on Akaike’s information criterion (AIC); "graphics" and "ggplot2" for graphic output; "rstatix" and "outliers" for detecting outliers.

Negative clearance rates, which are unrealistic, were discarded. For ingestion rates, the mean abundance of *Vorticella natans* was 14–fold higher than the target concentration of 1 cell mL^−1^ in the *Daphnia* experiments, resulting in highly deviating ingestion rates. Therefore, the dataset of the *Daphnia-Vorticella* experiment was removed from the analyses. We then identified further outliers for clearance rates and ingestion rates using boxplots methods with the package "rstatix" and Grubs’s test with the package "outliers". The resulting data set consisted of 78 (out of 79) values for clearance rates and 72 (out of 73) values for ingestion rates. Data were log_10_-transformed to improve their normality, when conducting regressions of rates vs. size. For the final analyses, we used linear models (LM) for log–transformed clearance rate and ingestion rate analyses (Supplementary Table S1). ANOVA and Tukey test were used to investigate pairwise differences between the treatments.

To test ciliate-specific susceptibility to predation in relation to ciliate size, we used power curve (*y* = *ax*^*b*^), exponential curve (*y* = *ae*^*bx*^), and ordinary least-squares linear regression (*y* = *kx* + *c*) analyses to fit both clearance rates and ingestion rates to ciliate size. The model with the lowest AIC score represents the model with the best fit (Suppl. Table S2), and models with differences (Δ, delta) in AIC scores > 2 were considered different^77^. Experimental results reported represent mean values ± standard deviation (SD). Results were considered significant if *p* was ≤ 0.05.

### Analysis of literature data

We first searched the literature for similar experimental work as presented in this study by using GoogleScholar and Scopus with ‘ciliate’, ‘copepod’, ‘*Daphnia*’, ‘top-down control’, experiments, ‘clearance rate’, ‘grazing’, ‘ingestion rate’, ‘lakes’, ‘freshwater’ and ‘marine’ as keywords. The search yielded 612 (Google Scholar), respectively 171 (Scopus) publications of which we selected 29 articles after removing dupicates. Secondly, we examined the citations and the reference lists of the selected papers in search of further, appropriate articles. This provided another 25 suitable publications. Selection criteria were realistic levels (i.e., close to in situ conditions) of prey, predator, and temperature. Further, we considered only ciliate species that were actively swimming, i.e. we discarded ciliates living attached to other organisms such as diatoms, cyanobacteria or copepods. The final selection of 54 articles represent 54 microcrustacean taxa and 59 defined ciliate species (see Data availability and Supplementary Information). The true number of ciliate species is likely higher since, in many cases, the taxonomic affiliation of the study ciliates remained undetermined. Graphed data were extracted from the original works by using WebPlotDigitizer (https://apps.automeris.io/wpd/).

We then performed a meta-analysis of the existing data with freshwater and marine prey and predators, following similar statistical procedures as outlined above for our experimental data (Suppl. Tables S3–S5). Size-specific ingestion rates were not tested because the database is meagre and heavily biased for marine calanoids. To account for temperature differences in the experimental studies reported in the literature, the data were normalised to a standard temperature (15 °C) assuming a Q_10_ value of 2.8^78^.

A list of data sources used in this study is provided in the Data sources section (see Supplementary Information).

## Supplementary Information

Supplementary Information.

## Data Availability

The experimental data and the dataset used for the meta-analysis are reported in the Supplementary Information.
