# Supplementary material for: Top-down control of planktonic ciliates by microcrustacean predators is stronger in lakes than in the ocean
Source: Sci Rep. 2022 Jun 22;12:10501. doi: 10.1038/s41598-022-14301-y (PMC9218117; doi:10.1038/s41598-022-14301-y)
Supplement: Supplementary file 1 — Supplementary Information. [file 41598_2022_14301_MOESM1_ESM.pdf]

SUPPLEMENTARY INFORMATION

Top-down control of planktonic ciliates by microcrustacean predators is stronger in lakes than in the ocean

Xiaoteng Lu[1] & Thomas Weisse*

University of Innsbruck, Research Department for Limnology, Mondseestr. 9, A-5310 Mondsee, Austria

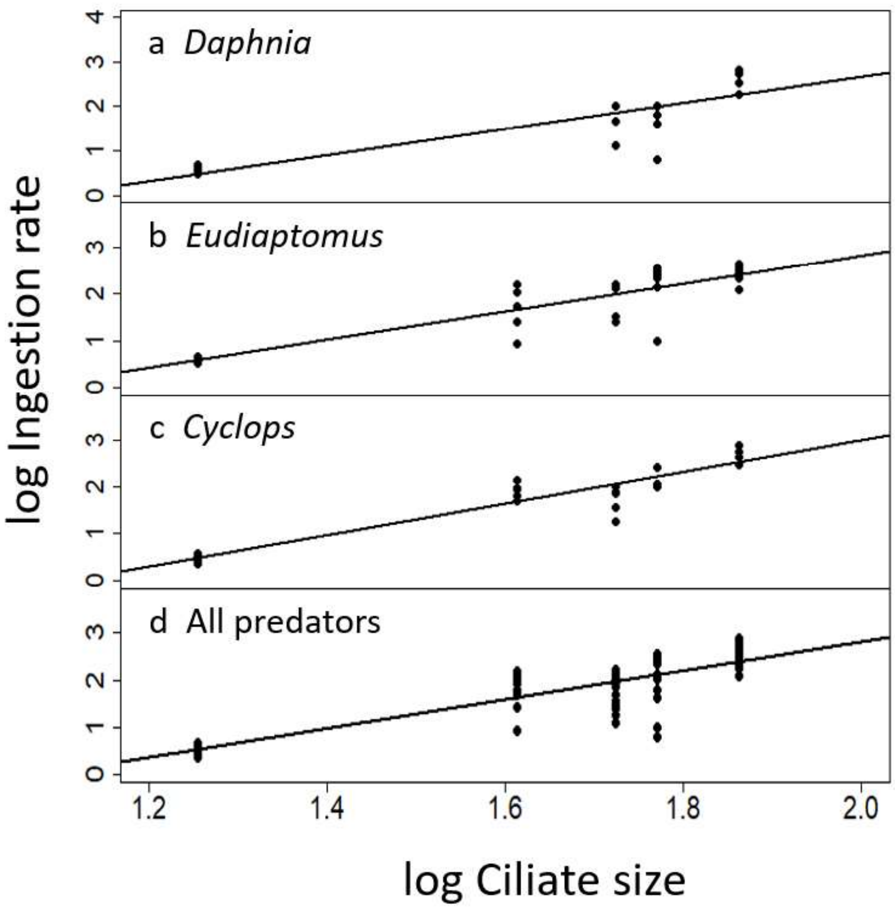

Supplementary Fig. S1. Linear regressions of log–transformed ingestion rates (logIR, in ng C ind^{-1} d^{-1}) of the three predators (a–c) and all predators combined (d) vs ciliate size.

Dataset used for the meta-analysis. Summary of the existing literature reporting clearance and ingestion rates from different functional groups of microcrustacean predators on ciliates at 15 °C. Where necessary, rates were normalised to 15 °C assuming a Q_{10} value of 2.8 [1]. Ciliate size in (µm); clearance rates (*CL*) in (mL individual^{-1} d^{-1}) and ingestion rates (*IR*) in (µgC individual^{-1} d^{-1}).

**Predator**	**Functional group**	**Ciliates**	**Ciliate size (µm)**	***CL***	***IR***	**Source**
Bosmina longirostris	FW cladocerans	*Tetrahymena pyriformis*	46	18		[2]
Bosmina longirostris	FW cladocerans	*Strobilidium gyrans*	43	4		[2]
Bosmina longirostris	FW cladocerans	*Colpidium striatum*	81	5		[2]
Bosmina longirostris	FW cladocerans	*Coleps octospinus*	100	6		[2]
Bosmina longirostris	FW cladocerans	*Paramecium tetraurelia*	120	6		[2]
Bosmina longirostris	FW cladocerans	*Euplotes eurystomus*	155	1		[2]
Ceriodaphnia dubia	FW cladocerans	Mixed ciliates		62		[3]
Daphnia carinata	FW cladocerans	Mixed ciliates		122		[4]
Daphnia carinata	FW cladocerans	Mixed ciliates		22		[5]
Daphnia carinata	FW cladocerans	Oligotrichs (> 20µm)		20		[5]
Daphnia galeata	FW cladocerans	*Askenasia* sp.	38	14		[6]
Daphnia galeata	FW cladocerans	*Halteria* sp.	28	5		[6]
Daphnia galeata	FW cladocerans	*Strombidium* sp. 'small'	46	19		[6]
Daphnia galeata	FW cladocerans	*Histiobalantium* sp.	50	16		[6]
Daphnia galeata	FW cladocerans	*Strombidium* sp. 'large'	65	29		[6]
Daphnia galeata	FW cladocerans	*Urotricha* sp.	30	12		[6]
Daphnia magna	FW cladocerans	*Cyclidium glaucoma*	30	6		[7]
Daphnia magna	FW cladocerans	*Paramecium caudatum*	200	2		[7]
Daphnia magna	FW cladocerans	*Tetrahymena pyriformis*	46	4		[2,8]
Daphnia pulex	FW cladocerans	*Coleps octospinus*	100	5		[2]
Daphnia pulex	FW cladocerans	*Coleps octospinus*	100	4		[2]
Daphnia pulex	FW cladocerans	*Colpidium striatum*	81	11		[2]
Daphnia pulex	FW cladocerans	*Colpidium striatum*	81	7		[2]
Daphnia pulex	FW cladocerans	*Euplotes eurystomus*	155	3		[2]
Daphnia pulex	FW cladocerans	*Euplotes eurystomus*	155	1		[2]
Daphnia pulex	FW cladocerans	*Paramecium tetraurelia*	120	5.9		[2]
Daphnia pulex	FW cladocerans	*Paramecium tetraurelia*	120	5.7		[2]
Daphnia pulex	FW cladocerans	*Strobitidium gyrans*	43	13		[2]
Daphnia pulex	FW cladocerans	*Strobitidium gyrans*	43	9		[2]
Daphnia pulex	FW cladocerans	*Tetrahymena pyriformis*	46	14.1		[2]
Daphnia pulex	FW cladocerans	*Tetrahymena pyriformis*	46	13.6		[2]
Daphnia rosea	FW cladocerans	Mixed ciliates		7		[9]
Daphnia sp.	FW cladocerans	*Histiobalantium bodamicum*	53	17	0.05	[10]
Daphnia sp.	FW cladocerans	*Rimostrombidium lacustris*	73	23	0.42	[10]
Daphnia sp.	FW cladocerans	*Strobilidium caudatum*	59	17	0.05	[10]
Daphnia sp.	FW cladocerans	*Urotricha* sp.	18	33	0	[10]
Daphnia sp.	FW cladocerans	*Vorticella natans*	41	18		[10]
Daphnia spp.	FW cladocerans	Mixed ciliates	33	9		[11]
Holopedium gibberum	FW cladocerans	Mixed ciliates	30	15		[9]
Acanthodiaptomus denticornis	FW calanoids	*Loxodes* sp.	228	12		[12]
Acanthodiaptomus denticornis	FW calanoids	*Paramecium aurelia*	114	64	2.26	[12]
Acanthodiaptomus denticornis	FW calanoids	*Paramecium aurelia*	114	19	4.59	[12]
Acanthodiaptomus denticornis	FW calanoids	*Paramecium aurelia*	114	79	3.82	[12]
Acanthodiaptomus denticornis	FW calanoids	*Paramecium aurelia*	114	39	11.97	[12]
Acanthodiaptomus denticornis	FW calanoids	*Paramecium caudatum*	124	65		[12]
Acanthodiaptomus denticornis	FW calanoids	*Tetrahymena corlisii*	32	31		[12]
Acanthodiaptomus denticornis	FW calanoids	*Tetrahymena corlisii*	32	34		[12]
Boeckella hamata	FW calanoids	Oligotrichs <20 µm		45		[5]

Boeckella hamata	FW calanoids	Oligotrichs >20 µm		68		[5]
Boeckella hamata	FW calanoids	Oligotrichs		342		[3]
Boeckella hamata	FW calanoids	Mixed ciliates		66		[5]
Boeckella hamata	FW calanoids	Mixed ciliates		101		[4]
Boeckella hamata	FW calanoids	Mixed ciliates		241		[3]
Boeckella hamata	FW calanoids	Mixed ciliates		177		[3]
Diaptomus minutus	FW calanoids	*Halteria* sp.	21	4	0.01	[13]
Diaptomus minutus	FW calanoids	*Halteria* sp.	21	5	0.01	[13]
Diaptomus minutus	FW calanoids	*Strobilidium velox*	61	14	0.08	[13,14]
Diaptomus minutus	FW calanoids	*Strobilidium velox*	61	36	0.09	[13]
Diaptomus minutus	FW calanoids	*Strobilidium velox*	61	15	0.18	[13]
Diaptomus minutus	FW calanoids	*Strobilidium velox*	61	7	0.06	[13]
Diaptomus minutus	FW calanoids	*Strobilidium* sp.	61	7	0.05	[13]
Diaptomus minutus	FW calanoids	*Strobilidium* sp.	50	28	0.01	[13]
Diaptomus minutus	FW calanoids	*Strobilidium* sp.	50	17	0.05	[13]
Diaptomus minutus	FW calanoids	*Strobilidium* sp.	50	30	0.11	[13]
Diaptomus minutus	FW calanoids	*Strobilidium* sp.	50	30	0.18	[13]
Diaptomus minutus	FW calanoids	Mixed ciliates (<10 µm)		19		[13]
Diaptomus minutus	FW calanoids	Mixed ciliates (<10 µm)		27		[13]
Diaptomus minutus	FW calanoids	Mixed ciliates (<10 µm)		17		[13]
Diaptomus minutus	FW calanoids	Mixed ciliates (>16 µm)		17		[13]
Diaptomus minutus	FW calanoids	Mixed ciliates (>16 µm)		19		[13]
Diaptomus minutus	FW calanoids	Mixed ciliates (>16 µm)		30		[13]
Diaptomus novamexicanus	FW calanoids	Mixed ciliates	30	12		[9]
Diaptomus pygmaeus	FW calanoids	*Cyclidium* sp.	17	2	0.43	[13]
Diaptomus pygmaeus	FW calanoids	*Strobilidium velox*	61	28	0.54	[13]
Diaptomus pygmaeus	FW calanoids	*Strobilidium velox*	61	24	0.18	[13]
Diaptomus pygmaeus	FW calanoids	*Strobilidium velox*	61	31	0.25	[13]
Diaptomus pygmaeus	FW calanoids	*Strobilidium* sp.	50	42	0.03	[13]
Diaptomus pygmaeus	FW calanoids	*Strobilidium* sp.	50	17	0.02	[13]
Diaptomus pygmaeus	FW calanoids	Mixed ciliates (<10 µm)		13	0.003	[13]
Epischura lacustris	FW calanoids	*Strobilidium acutum*	50	267	1.52	[13]
Epischura lacustris	FW calanoids	*Strobilidium velox*	61	255	1.20	[13]
Epischura lacustris	FW calanoids	*Strobilidium velox*	61	102	0.60	[13]
Epischura lacustris	FW calanoids	*Strobilidium velox*	61	120	0.96	[13]
Epischura lacustris	FW calanoids	*Strobilidium velox*	61	311	1.06	[13]
Epischura lacustris	FW calanoids	*Strobilidium* sp.	50	147	0.52	[13]
Eudiaptomus gracilis	FW calanoids	*Askenasia* sp. 2	24	19		[54]
Eudiaptomus gracilis	FW calanoids	*Askenasia* sp. 23	45	29		[54]
Eudiaptomus gracilis	FW calanoids	Small mixed ciliates	18	24		[54]
Eudiaptomus gracilis	FW calanoids	*Balanion planctonicum*	20	12		[54]
Eudiaptomus gracilis	FW calanoids	*Codonella* spp.	70	34		[54]
Eudiaptomus gracilis	FW calanoids	*Coleps* spp.	53	9		[54]
Eudiaptomus gracilis	FW calanoids	*Cyclidium* spp.	21	13		[54]
Eudiaptomus gracilis	FW calanoids	*Didinium nasutum*	55	3		[54]
Eudiaptomus gracilis	FW calanoids	*Didinium* sp.	45	5		[54]
Eudiaptomus gracilis	FW calanoids	*Halteria* sp. 2 / *Strobilidium*	26	22		[54]
Eudiaptomus gracilis	FW calanoids	*Histiobalantium* sp. 1	27	19		[54]
Eudiaptomus gracilis	FW calanoids	*Histiobalantium* sp. 2	45	21		[54]
Eudiaptomus gracilis	FW calanoids	*Histiobalantium* sp. 3	60	3		[54]
Eudiaptomus gracilis	FW calanoids	*Lacrymaria* spp.		7		[54]
Eudiaptomus gracilis	FW calanoids	*Lagynophrya* sp. 1		13		[54]
Eudiaptomus gracilis	FW calanoids	*Lagynophrya* sp. 2	90	5		[54]
Eudiaptomus gracilis	FW calanoids	*Paradileptus* spp.	135	18		[54]
Eudiaptomus gracilis	FW calanoids	*Pelagohalteria viridis*	23	1		[54]
Eudiaptomus gracilis	FW calanoids	*Pelagostrombidium* sp.1	50	29		[54]
Eudiaptomus gracilis	FW calanoids	*Pelagostrombidium mirabile*	68	19		[54]
Eudiaptomus gracilis	FW calanoids	*Rimostrombidium lacustris*	73	15		[54]
Eudiaptomus gracilis	FW calanoids	*Staurophyra* spp.	40	14		[54]
Eudiaptomus gracilis	FW calanoids	*Stichotricha* spp.		8		[54]
Eudiaptomus gracilis	FW calanoids	*Tintinnidium* sp. 1	45	46		[54]
Eudiaptomus gracilis	FW calanoids	*Tintinnidium* sp. 2	70	44		[54]

Eudiaptomus gracilis	FW calanoids	*Tintinnidium* sp. 3	86	7		[54]
Eudiaptomus gracilis	FW calanoids	*Urotricha furcata*	25	21		[54]
Eudiaptomus gracilis	FW calanoids	*Urotricha* sp. 2	35	49		[54]
Eudiaptomus gracilis	FW calanoids	*Urotricha* sp. 3	55	27		[54]
Eudiaptomus gracilis	FW calanoids	*Urotricha* sp. 4	65	22		[54]
Eudiaptomus gracilis	FW calanoids	*Vorticella* sp. 'free'	35	21		[54]
Eudiaptomus gracilis	FW calanoids	*Vorticella* sp.'free' 2	50	21		[54]
Eudiaptomus graciloides	FW calanoids	Mixed ciliates	33	37		[11]
Eudiaptomus copepodites	FW calanoids	Mixed ciliates	33	26		[11]
Eudiaptomus sp.	FW calanoids	*Histiobalantium bodamicum*	53	16	0.10	[10]
Eudiaptomus sp.	FW calanoids	*Rimostrombidium lacustris*	73	10	0.29	[10]
Eudiaptomus sp.	FW calanoids	*Strobilidium caudatum*	59	22	0.21	[10]
Eudiaptomus sp.	FW calanoids	*Urotricha* sp.	18	39	0.004	[10]
Eudiaptomus sp.	FW calanoids	*Vorticella natans*	41	24	0.07	[10]
Cyclops abyssorum	FW cyclopoids	*Askenasia volvox*	40	22	0.14	[15]
Cyclops abyssorum	FW cyclopoids	*Askenasia* sp.	38	14		[6]
Cyclops abyssorum	FW cyclopoids	*Coleps hirtus*	60	12	0.50	[15]
Cyclops abyssorum	FW cyclopoids	*Halteria grandinella*	22	4	0.02	[15]
Cyclops abyssorum	FW cyclopoids	*Halteria* sp.	28	20		[6]
Cyclops abyssorum	FW cyclopoids	*Halteria* sp.	28	5		[6]
Cyclops abyssorum	FW cyclopoids	*Histiobalantium* sp.	50	18		[6]
Cyclops abyssorum	FW cyclopoids	*Strobilidium velox*	50	33	4.03	[15]
Cyclops abyssorum	FW cyclopoids	*Stokesia vernalis*	120	14	0.22	[15]
Cyclops abyssorum	FW cyclopoids	*Strombidium* sp. 'small'	46	24		[6]
Cyclops abyssorum	FW cyclopoids	*Strombidium* sp. 'small'	46	11		[6]
Cyclops abyssorum	FW cyclopoids	*Strombidium* sp. 'large'	65	55		[6]
Cyclops abyssorum	FW cyclopoids	*Urotricha* sp.	30	4		[6]
Cyclops kolensis	FW cyclopoids	*Askenasia volvox*	40	54	0.46	[15]
Cyclops kolensis	FW cyclopoids	*Coleps hirtus*	60	10	0.41	[15]
Cyclops kolensis	FW cyclopoids	*Halteria grandinella*	22	7	0.08	[15]
Cyclops kolensis	FW cyclopoids	*Strobilidium velox*	50	159	19.75	[15]
Cyclops kolensis	FW cyclopoids	*Stokesia vernalis*	120	362	1.44	[15]
Cyclops sp.	FW cyclopoids	*Histiobalantium bodamicum*	53	21	0.06	[10]
Cyclops sp.	FW cyclopoids	*Rimostrombidium lacustris*	73	23	0.51	[10]
Cyclops sp.	FW cyclopoids	*Strobilidium caudatum*	59	22	0.16	[10]
Cyclops sp.	FW cyclopoids	*Urotricha* sp.	18	23	0.003	[10]
Cyclops sp.	FW cyclopoids	*Vorticella natans*	41	25	0.09	[10]
Diacyclops bicuspidatus thomasi	FW cyclopoids	Mixed ciliates	30	40		[9]
Mesocyclops thermocyclopoides	FW cyclopoids	*Paramecium caudatum*	176	81		[16]
Mesocyclops thermocyclopoides	FW cyclopoids	*Pseudourostyla levis*	214	5		[16]
Mesocyclops thermocyclopoides	FW cyclopoids	*Stylonychia notophora*	88	67		[16]
Mesocyclops thermocyclopoides	FW cyclopoids	Mixed ciliates	159	54		[16]
Various cyclopoids	FW cyclopoids	Mixed ciliates	33	17		[11]
Evadne spinifera	Marine cladocerans	Mixed ciliates		24	0.15	[17]
Evadne spinifera	Marine cladocerans	Mixed ciliates		4	0.01	[17]
Penilia avirostris	Marine cladocerans	Mixed ciliates		11	0.33	[17]
Penilia avirostris	Marine cladocerans	Mixed ciliates		8	0.01	[17]
Penilia avirostris	Marine cladocerans	Mixed ciliates		8	0.19	[17]
Penilia avirostris	Marine cladocerans	Mixed ciliates		3	0.02	[17]
Penilia avirostris	Marine cladocerans	Mixed ciliates		2		[18]
Penilia avirostris	Marine cladocerans	Mixed ciliates		5		[18]
Penilia avirostris	Marine cladocerans	Mixed ciliates		1		[18]
Podon sp.	Marine cladocerans	Mixed ciliates		30	0.11	[17]
Acartia clausi	Marine calanoids	*Favella taraikaensis*	210	47	0.40	[12,19]
Acartia clausi	Marine calanoids	*Helicostomella fusiformis*	110	17	0.30	[12,19]
Acartia clausi	Marine calanoids	*Helicostomella fusiformis*	110	7.4	1.30	[12,19]
Acartia clausi	Marine calanoids	*Lohmanniella oviformis*	18	197		[20]
Acartia clausi	Marine calanoids	*Strombidium conicum*	48	62		[20]
Acartia clausi	Marine calanoids	*Strombidium sulcatum*	30	283	400	[12,17]

Acartia clausi	Marine calanoids	*Strombidium sulcatum*	30	183	123	[12,17]
Acartia clausi	Marine calanoids	*Strombidium vestitum*	23	58		[20]
Acartia clausi	Marine calanoids	*Strombidium* sp.	40	261		[20]
Acartia clausi	Marine calanoids	Choreotrichs (< 20 µm)		49	0.7	[21]
Acartia clausi	Marine calanoids	Choreotrichs (< 20 µm)		88	0.3	[21]
Acartia clausi	Marine calanoids	Choreotrichs (< 20 µm)		89	0.4	[21]
Acartia clausi	Marine calanoids	Choreotrichs (< 20 µm)		29	0.1	[21]
Acartia clausi	Marine calanoids	Choreotrichs (< 20 µm)		29	0.1	[21]
Acartia clausi	Marine calanoids	Choreotrichs (< 20 µm)		53	0.2	[21]
Acartia clausi	Marine calanoids	Choreotrichs (< 20 µm)		35	0.1	[21]
Acartia clausi	Marine calanoids	Choreotrichs (< 20 µm)		20	0.1	[21]
Acartia clausi	Marine calanoids	Choreotrichs (< 20 µm)		100	0.04	[21]
Acartia clausi	Marine calanoids	Choreotrichs (< 20 µm)		48	0.04	[21]
Acartia clausi	Marine calanoids	Choreotrichs (< 20 µm)		15	0.03	[21]
Acartia clausi	Marine calanoids	Mixed ciliates	11	28		[22]
Acartia clausi	Marine calanoids	Mixed ciliates	15	32		[22]
Acartia clausi	Marine calanoids	Mixed ciliates	19	34		[22]
Acartia clausi	Marine calanoids	Mixed ciliates	26	49		[22]
Acartia clausi	Marine calanoids	Mixed ciliates	31	53		[22]
Acartia clausi	Marine calanoids	Mixed ciliates	36	47		[22]
Acartia clausi	Marine calanoids	Mixed ciliates	47	50		[22]
Acartia clausi	Marine calanoids	Mixed ciliates		21		[22]
Acartia hudsonica	Marine calanoids	*Eutintinnus pectinis*	150	3		[23]
Acartia hudsonica	Marine calanoids	*Eutintinnus pectinis*	150	3		[23]
Acartia hudsonica	Marine calanoids	*Eutintinnus pectinis*	150	3		[23]
Acartia hudsonica	Marine calanoids	*Eutintinnus pectinis*	150	5		[23]
Acartia tonsa	Marine calanoids	*Balanion* sp.	34	60	0.54	[24]
Acartia tonsa	Marine calanoids	*Balanion* sp.	34	63	0.51	[24]
Acartia tonsa	Marine calanoids	*Favella panamensis*	265	31	1.37	[25]
Acartia tonsa	Marine calanoids	*Favella* sp.	150	32	1.76	[24]
Acartia tonsa	Marine calanoids	*Favella* sp.	150	4	0.19	[24]
Acartia tonsa	Marine calanoids	*Favella* sp.	150	119	1.09	[24]
Acartia tonsa	Marine calanoids	*Favella* sp.	150	149	0.68	[24]
Acartia tonsa	Marine calanoids	*Favella* sp.	150	115	0.19	[26]
Acartia tonsa	Marine calanoids	*Favella* sp.	150	214		[26]
Acartia tonsa	Marine calanoids	*Mesodinium rubrum*	43	15		[27]
Acartia tonsa	Marine calanoids	*Strobilidium spiralis*	65	90		[27]
Acartia tonsa	Marine calanoids	*Strombidium reticulatum*	43	34		[27]
Acartia tonsa	Marine calanoids	*Strobilidium* sp.	52	44	0.51	[24]
Acartia tonsa	Marine calanoids	*Strobilidium* sp.	52	27	0.41	[24]
Acartia tonsa	Marine calanoids	*Strobilidium* sp.	52	36	0.46	[24]
Acartia tonsa	Marine calanoids	*Tintinnopsis tubulosa*	148	56	0.27	[25]
Acartia tonsa	Marine calanoids	*Tintinnopsis* sp.	65	17		[24]
Acartia tonsa	Marine calanoids	*Tintinnopsis* sp.	65	40		[24]
Acartia tonsa	Marine calanoids	*Urotricha* sp.	12	39	0.004	[24]
Acartia (Acanthacartia) tonsa	Marine calanoids	Mixed ciliates		135	2.55	[28]
Acartia (Acanthacartia) tonsa	Marine calanoids	Mixed ciliates		3	9.69	[28]
Acartia tonsa	Marine calanoids	Mixed ciliates	62	12	0.06	[29]
Acartia tonsa	Marine calanoids	Mixed ciliates	62	188	0.29	[29]
Acartia tonsa	Marine calanoids	Mixed ciliates	62	211	0.38	[29]
Acartia spp.	Marine calanoids	Aloricate ciliates		22	0.04	[30]
Acartia spp.	Marine calanoids	Aloricate ciliates		3	0.05	[30]
Acartia spp.	Marine calanoids	Aloricate ciliates		19	0.04	[30]
Acartia spp.	Marine calanoids	Aloricate ciliates		29	0.12	[30]
Acartia spp.	Marine calanoids	Aloricate ciliates		15	0.01	[30]
Acartia spp.	Marine calanoids	Aloricate ciliates		17	0.01	[30]
Acartia spp.	Marine calanoids	Loricate ciliates		33	0.03	[30]
Acartia spp.	Marine calanoids	Loricate ciliates		6		[30]
Acartia spp.	Marine calanoids	Loricate ciliates		39	0.09	[30]
Acartia spp.	Marine calanoids	Loricate ciliates		44	0.10	[30]
Acartia spp.	Marine calanoids	Loricate ciliates		18	0.02	[30]
Acartia spp.	Marine calanoids	Loricate ciliates		40	0.48	[30]

Acartia spp.	Marine calanoids	Loricate ciliates		10	0.08	[30]
Acartia spp.	Marine calanoids	Mixed ciliates		97		[31]
Acartia spp.	Marine calanoids	Mixed ciliates (16-20 µm)		42		[32]
Acartia spp.	Marine calanoids	Mixed ciliates (21-30 µm)		66		[32]
Acartia spp.	Marine calanoids	Mixed ciliates (31-45 µm)		119		[32]
Aetideus divergens	Marine calanoids	Mixed ciliates		394		[28]
Calanoides acutus	Marine calanoids	Mixed ciliates		190		[33]
Calanus finmarchicus	Marine calanoids	*Myrionecta rubra*		1904		[34]
Calanus finmarchicus	Marine calanoids	Mixed ciliates	30	20	2.94	[35]
Calanus finmarchicus	Marine calanoids	Mixed ciliates	30	15	3.04	[35]
Calanus finmarchicus	Marine calanoids	Mixed ciliates	30	25	2.98	[35]
Calanus finmarchicus	Marine calanoids	Mixed ciliates	30	44	5.43	[35]
Calanus finmarchicus	Marine calanoids	Mixed ciliates	30	56	10.71	[35]
Calanus finmarchicus	Marine calanoids	Mixed ciliates (>30 µm)		469	0.17	[36]
Calanus finmarchicus	Marine calanoids	Mixed ciliates (>30 µm)		837	2.68	[36]
Calanus finmarchicus	Marine calanoids	Mixed ciliates (>30 µm)		328	9.04	[36]
Calanus finmarchicus	Marine calanoids	Mixed ciliates (>30 µm)		321	3.85	[36]
Calanus finmarchicus	Marine calanoids	Mixed ciliates (<30 µm)		301	6.53	[36]
Calanus finmarchicus	Marine calanoids	Mixed ciliates (<30 µm)		241	7.20	[36]
Calanus finmarchicus	Marine calanoids	Mixed ciliates (<30 µm)		59	2.01	[36]
Calanus finmarchicus	Marine calanoids	Mixed ciliates (<30 µm)		60	1.00	[36]
Calanus finmarchicus	Marine calanoids	Mixed ciliates		20	0.25	[36]
Calanus finmarchicus	Marine calanoids	Mixed ciliates		217	3.54	[36]
Calanus finmarchicus	Marine calanoids	Mixed ciliates		129	0.51	[36]
Calanus finmarchicus	Marine calanoids	Mixed ciliates		200	2.27	[36]
Calanus finmarchicus	Marine calanoids	Mixed ciliates		154	0.51	[36]
Calanus finmarchicus	Marine calanoids	Mixed ciliates		642	0.76	[36]
Calanus finmarchicus	Marine calanoids	Mixed ciliates		619	1.73	[36]
Calanus finmarchicus	Marine calanoids	Mixed ciliates		1078	16.67	[36]
Calanus finmarchicus	Marine calanoids	Oligotrich ciliates (< 20µm)		698		[34]
Calanus finmarchicus	Marine calanoids	Oligotrich ciliates (< 20µm)		952		[34]
Calanus finmarchicus	Marine calanoids	Oligotrichous ciliates (>		1689		[34]
Calanus glacialis	Marine calanoids	Oligotrichous ciliates (<		1828		[34]
Calanus helgolandicus	Marine calanoids	*Myrionecta rubra*	40	783	0.30	[21]
Calanus helgolandicus	Marine calanoids	*Myrionecta rubra*	40	653	0.30	[21]
Calanus helgolandicus	Marine calanoids	*Myrionecta rubra*	40	626	0.30	[21]
Calanus helgolandicus	Marine calanoids	*Myrionecta rubra*	40	1164	0.30	[21]
Calanus helgolandicus	Marine calanoids	*Myrionecta rubra*	40	1347	0.30	[21]
Calanus helgolandicus	Marine calanoids	*Myrionecta rubra*	40	1099	0.30	[21]
Calanus helgolandicus	Marine calanoids	*Myrionecta rubra*	40	1347	0.30	[21]
Calanus helgolandicus	Marine calanoids	*Myrionecta rubra*	40	799	0.30	[21]
Calanus helgolandicus	Marine calanoids	Aloricate choreotrichs		177	0.40	[21]
Calanus helgolandicus	Marine calanoids	Aloricate choreotrichs		330	0.40	[21]
Calanus helgolandicus	Marine calanoids	Aloricate choreotrichs		229	0.40	[21]
Calanus helgolandicus	Marine calanoids	Aloricate choreotrichs		188	0.40	[21]
Calanus helgolandicus	Marine calanoids	Aloricate choreotrichs		295	0.40	[21]
Calanus helgolandicus	Marine calanoids	Aloricate choreotrichs		416	0.40	[21]
Calanus helgolandicus	Marine calanoids	Aloricate choreotrichs		263	0.40	[21]
Calanus helgolandicus	Marine calanoids	Aloricate choreotrichs		273	0.40	[21]
Calanus helgolandicus	Marine calanoids	Choreotrichs (< 20 µm)		2353	0.20	[21]
Calanus helgolandicus	Marine calanoids	Choreotrichs (< 20 µm)		269	0.60	[21]
Calanus helgolandicus	Marine calanoids	Choreotrichs (< 20 µm)		217	0.10	[21]
Calanus helgolandicus	Marine calanoids	Choreotrichs (< 20 µm)		354	4.80	[21]
Calanus helgolandicus	Marine calanoids	Choreotrichs (< 20 µm)		278	2.60	[21]
Calanus helgolandicus	Marine calanoids	Choreotrichs (< 20 µm)		347	0.20	[21]
Calanus helgolandicus	Marine calanoids	Choreotrichs (< 20 µm)		19	0.02	[21]
Calanus helgolandicus	Marine calanoids	Choreotrichs (< 20 µm)		104	0.10	[21]
Calanus helgolandicus	Marine calanoids	Choreotrichs (> 20 µm)		25	0.02	[21]
Calanus helgolandicus	Marine calanoids	Choreotrichs (> 20 µm)		151	0.40	[21]
Calanus helgolandicus	Marine calanoids	Choreotrichs (> 20 µm)		269	1.20	[21]
Calanus helgolandicus	Marine calanoids	Choreotrichs (> 20 µm)		427	1.70	[21]
Calanus helgolandicus	Marine calanoids	Choreotrichs (> 20 µm)		328	0.50	[21]

Calanus helgolandicus	Marine calanoids	Choreotrichs (> 20 µm)		140	0.10	[21]
Calanus helgolandicus	Marine calanoids	Mixed ciliates		95	7.6	[37]
Calanus helgolandicus	Marine calanoids	Mixed ciliates			54.3	[28]
Calanus helgolandicus	Marine calanoids	Mixed ciliates			54.6	[28]
Calanus helgolandicus	Marine calanoids	Mixed ciliates			65.1	[28]
Calanus helgolandicus	Marine calanoids	Mixed ciliates		12	0.2	[36]
Calanus helgolandicus	Marine calanoids	Mixed ciliates		205	4.1	[36]
Calanus helgolandicus	Marine calanoids	Mixed ciliates		234	2.0	[36]
Calanus helgolandicus	Marine calanoids	Mixed ciliates		242	3.2	[36]
Calanus helgolandicus	Marine calanoids	Mixed ciliates		122	0.8	[36]
Calanus helgolandicus	Marine calanoids	Mixed ciliates		596	20.4	[36]
Calanus helgolandicus	Marine calanoids	Mixed ciliates		189	0.3	[36]
Calanus helgolandicus	Marine calanoids	Mixed ciliates		506	7.4	[36]
Calanus hyperboreus	Marine calanoids	Oligotrichous ciliates (<		2245		[34]
Calanus hyperboreus	Marine calanoids	Oligotrichous ciliates (<		2150		[34]
Calanus hyperboreus	Marine calanoids	Oligotrichous ciliates (>		8634		[34]
Calanus hyperboreus	Marine calanoids	Oligotrichous ciliates (>		1290		[34]
Calanus pacificus	Marine calanoids	Mixed ciliates		283	38.2	[28]
Calanus pacificus	Marine calanoids	Mixed ciliates		409	31.7	[28]
Calanus pacificus	Marine calanoids	Mixed ciliates		540	36.0	[28]
Calanus pacificus	Marine calanoids	Mixed ciliates		1301	10.9	[38]
Calanus pacificus	Marine calanoids	Mixed ciliates		1233	10.9	[38]
Calanus pacificus	Marine calanoids	Mixed ciliates		928	10.9	[38]
Calanus pacificus	Marine calanoids	Mixed ciliates		506	10.9	[38]
Calanus simillimus	Marine calanoids	Mixed ciliates		154		[33]
Calanus spp.	Marine calanoids	Mixed ciliates (16-20 µm)		218		[32]
Calanus spp.	Marine calanoids	Mixed ciliates (21-30 µm)		360		[32]
Calanus spp.	Marine calanoids	Mixed ciliates (31-45 µm)		497		[32]
Centropages abdominalis	Marine calanoids	Mixed ciliates		285	0.8	[38]
Centropages abdominalis	Marine calanoids	Mixed ciliates		277	0.8	[38]
Centropages abdominalis	Marine calanoids	Mixed ciliates		277	0.8	[38]
Centropages abdominalis	Marine calanoids	Mixed ciliates		137	0.3	[38]
Centropages abdominalis	Marine calanoids	Mixed ciliates		92	0.3	[38]
Centropages abdominalis	Marine calanoids	Mixed ciliates		48	0.3	[38]
Centropages abdominalis	Marine calanoids	Mixed ciliates		253	1.5	[38]
Centropages abdominalis	Marine calanoids	Mixed ciliates		261	1.5	[38]
Centropages brachiatus	Marine calanoids	Mixed ciliates	62	186	0.45	[29]
Centropages brachiatus	Marine calanoids	Mixed ciliates	62	241	0.07	[29]
Centropages chierchiae	Marine calanoids	Mixed ciliates		149	11.3	[37]
Centropages chierchiae	Marine calanoids	Mixed ciliates (16-20 µm)		76		[32]
Centropages chierchiae	Marine calanoids	Mixed ciliates (21-30 µm)		107		[32]
Centropages chierchiae	Marine calanoids	Mixed ciliates (31-45 µm)		236		[32]
Centropages hamatus	Marine calanoids	Mixed ciliates	11	31		[22]
Centropages hamatus	Marine calanoids	Mixed ciliates	15	41		[22]
Centropages hamatus	Marine calanoids	Mixed ciliates	19	57		[22]
Centropages hamatus	Marine calanoids	Mixed ciliates	26	73		[22]
Centropages hamatus	Marine calanoids	Mixed ciliates	31	92		[22]
Centropages hamatus	Marine calanoids	Mixed ciliates	36	102		[22]
Centropages hamatus	Marine calanoids	Mixed ciliates	47	124		[22]
Centropages hamatus	Marine calanoids	Mixed ciliates		28		[39]
Centropages typicus	Marine calanoids	*Strombidium sulcatum*	30	520	831	[12,40]
Centropages typicus	Marine calanoids	*Strombidium sulcatum*	30	337	137	[12,40]
Clausocalanus lividus	Marine calanoids	Mixed ciliates		121	15.9	[28]
Clausocalanus lividus	Marine calanoids	Mixed ciliates		210	15.4	[28]
Clausocalanus spp.	Marine calanoids	Mixed ciliates		55	0.03	[32]
Clausocalanus spp.	Marine calanoids	Mixed ciliates		16	0.08	[32]
Clausocalanus spp.	Marine calanoids	Mixed ciliates (16-20 µm)		50		[32]
Clausocalanus spp.	Marine calanoids	Mixed ciliates (21-30 µm)		38		[32]
Clausocalanus spp.	Marine calanoids	Mixed ciliates (31-45 µm)		36		[32]
Eucalanus pileatus	Marine calanoids	Mixed ciliates		72	1.01	[41]
Eucalanus pileatus	Marine calanoids	Mixed ciliates		54	0.76	[41]
Eucalanus pileatus	Marine calanoids	Mixed ciliates		67	0.87	[41]

Eucalanus pileatus	Marine calanoids	Mixed ciliates		75	1.32	[41]
Eucalanus pileatus	Marine calanoids	Mixed ciliates		55	1.06	[41]
Eurytemora affinis	Marine calanoids	*Uronema* sp.	17	3	3.55	[12,42]
Metridia lucens	Marine calanoids	Mixed ciliates		78		[33]
M. lucens/C lausocalanus	Marine calanoids	Mixed ciliates		318		[33]
Neocalanus cristatus	Marine calanoids	*Mesodinium* spp		2167		[43]
Neocalanus cristatus	Marine calanoids	Ciliates (<20 µm)		848		[43]
Neocalanus cristatus	Marine calanoids	Ciliates (>20 µm)		1697		[43]
Neocalanus cristatus	Marine calanoids	Mixed ciliates		1183	5.42	[43]
Neocalanus plumchrus	Marine calanoids	Mixed ciliates		655		[44,45]
Neocalanus tonsus	Marine calanoids	Mixed ciliates		105		[46]
Neocalanus tonsus	Marine calanoids	Mixed ciliates		105		[46]
Neocalanus tonsus	Marine calanoids	Mixed ciliates		264		[33]
Neocalanus spp.	Marine calanoids	Ciliates (< 20 µm)		816		[47]
Neocalanus spp.	Marine calanoids	Ciliates (20-30 µm)		1668		[47]
Neocalanus spp.	Marine calanoids	Ciliates (> 30 µm)		1587		[47]
Neocalanus spp.	Marine calanoids	Ciliates (< 20 µm)		404		[47]
Neocalanus spp.	Marine calanoids	Ciliates (20-30 µm)		1222		[47]
Neocalanus spp.	Marine calanoids	Ciliates (> 30 µm)		776		[47]
Neocalanus spp.	Marine calanoids	Ciliates (< 20 µm)		525		[47]
Neocalanus spp.	Marine calanoids	Ciliates (20-30 µm)		721		[47]
Neocalanus spp.	Marine calanoids	Ciliates (> 30 µm)		1111		[47]
Neocalanus spp.	Marine calanoids	Ciliates (< 20 µm)		130		[47]
Neocalanus spp.	Marine calanoids	Ciliates (20-30 µm)		547		[47]
Neocalanus spp.	Marine calanoids	Ciliates (30-40 µm)		644		[47]
Neocalanus spp.	Marine calanoids	Ciliates (40-50 µm)		529		[47]
Neocalanus spp.	Marine calanoids	Ciliates (> 50 µm)		612		[47]
Neocalanus spp.	Marine calanoids	Ciliates (< 20 µm)		94		[47]
Neocalanus spp.	Marine calanoids	Ciliates (20-30 µm)		476		[47]
Neocalanus spp.	Marine calanoids	Ciliates (> 30 µm)		87		[47]
Neocalanus spp.	Marine calanoids	Ciliates (< 20 µm)		261		[47]
Neocalanus spp.	Marine calanoids	Ciliates (20-30 µm)		282		[47]
Neocalanus spp.	Marine calanoids	Ciliates (30-40 µm)		281		[47]
Neocalanus spp.	Marine calanoids	Ciliates (> 50 µm)		591		[47]
Neocalanus spp.	Marine calanoids	Ciliates (< 20 µm)		134		[47]
Neocalanus spp.	Marine calanoids	Ciliates (20-30 µm)		191		[47]
Neocalanus spp.	Marine calanoids	Ciliates (30-40 µm)		366		[47]
Neocalanus spp.	Marine calanoids	Ciliates (40-50 µm)		428		[47]
Neocalanus spp.	Marine calanoids	Ciliates (> 50 µm)		311		[47]
Paracartia grani	Marine calanoids	Mixed ciliates			7.89	[28]
Paracalanus parvus	Marine calanoids	Mixed ciliates		235	0.09	[29]
Paracalanus parvus	Marine calanoids	Mixed ciliates		186	0.06	[29]
Paracalanus parvus	Marine calanoids	Mixed ciliates		54	0.28	[17]
Paracalanus parvus	Marine calanoids	Mixed ciliates		32	0.03	[17]
Paracalanus parvus	Marine calanoids	Mixed ciliates		11	0.33	[17]
Paracalanus parvus	Marine calanoids	Mixed ciliates		11	0.21	[17]
Paracalanus sp.	Marine calanoids	Ciliates (15-20µm)	18	14	0.02	[48]
Paracalanus sp.	Marine calanoids	Ciliates (15-20µm)	18	15	0.02	[48]
Paracalanus sp.	Marine calanoids	Ciliates (20-40µm)	30	11	0.01	[48]
Paracalanus sp.	Marine calanoids	Ciliates (>40µm)	50	22	0.09	[48]
Paracalanus sp.	Marine calanoids	Ciliates (15-20µm)	18	8	0.01	[48]
Para-pseudocalanus spp.	Marine calanoids	*Myrionecta rubra*	40	173	0.03	[21]
Para-pseudocalanus spp.	Marine calanoids	*Myrionecta rubra*	40	101	0.03	[21]
Para-pseudocalanus spp.	Marine calanoids	*Myrionecta rubra*	40	82	0.03	[21]
Para-pseudocalanus spp.	Marine calanoids	*Myrionecta rubra*	40	8	0.03	[21]
Para-pseudocalanus spp.	Marine calanoids	*Myrionecta rubra*	40	89	0.03	[21]
Para-pseudocalanus spp.	Marine calanoids	*Myrionecta rubra*	40	105	0.03	[21]
Para-pseudocalanus spp.	Marine calanoids	*Myrionecta rubra*	40	90	0.03	[21]
Para-pseudocalanus spp.	Marine calanoids	*Myrionecta rubra*	40	50	0.03	[21]
Para-pseudocalanus spp.	Marine calanoids	Aloricate choreotrichs		90	0.04	[21]
Para-pseudocalanus spp.	Marine calanoids	Aloricate choreotrichs		101	0.04	[21]
Para-pseudocalanus spp.	Marine calanoids	Aloricate choreotrichs		46	0.04	[21]

Para-pseudocalanus spp.	Marine calanoids	Aloricate choreotrichs		6	0.04	[21]
Para-pseudocalanus spp.	Marine calanoids	Aloricate choreotrichs		45	0.04	[21]
Para-pseudocalanus spp.	Marine calanoids	Aloricate choreotrichs		23	0.04	[21]
Para-pseudocalanus spp.	Marine calanoids	Aloricate choreotrichs		10	0.04	[21]
Para-pseudocalanus spp.	Marine calanoids	Aloricate choreotrichs		31	0.04	[21]
Para-pseudocalanus spp.	Marine calanoids	Mixed ciliates (16-20 µm)		61		[32]
Para-pseudocalanus spp.	Marine calanoids	Mixed ciliates (21-30 µm)		90		[32]
Para-pseudocalanus spp.	Marine calanoids	Mixed ciliates (31-45 µm)		98		[32]
Pseudocalanus sp.	Marine calanoids	*Lohmanniella oviformis*	18	425		[20]
Pseudocalanus sp.	Marine calanoids	*Strombidium conicum*	48	210		[20]
Pseudocalanus sp.	Marine calanoids	*Strombidium sp.*	40	402		[20]
Pseudocalanus sp.	Marine calanoids	Mixed ciliates		193	7.7	[38]
Pseudocalanus sp.	Marine calanoids	Mixed ciliates		189	7.7	[38]
Pseudocalanus sp.	Marine calanoids	Mixed ciliates		297	7.7	[38]
Pseudocalanus sp.	Marine calanoids	Mixed ciliates		289	7.7	[38]
Temora longicornis	Marine calanoids	*Strombidium elegans*	30	33		[49]
Temora longicornis	Marine calanoids	*Strombidium sulcatum*	30	92		[49]
Temora longicornis	Marine calanoids	Mixed ciliates		110	8.6	[37]
Temora stylifera	Marine calanoids	Mixed ciliates		51	0.03	[17]
Temora stylifera	Marine calanoids	Mixed ciliates		10	0.06	[17]
Tortanus sp.	Marine calanoids	Mixed ciliates		84	1.84	[28]
Tortanus sp.	Marine calanoids	Mixed ciliates		44	1.39	[28]
Oithona davisae	Marine cyclopoids	*Strombidium sulcatum*	29	10	0.07	[50]
Oithona davisae	Marine cyclopoids	Mixed ciliates		3	0.12	[28]
Oithona davisae	Marine cyclopoids	Mixed ciliates		5	0.14	[28]
Oithona davisae	Marine cyclopoids	Mixed ciliates		1	0.06	[28]
Oithona nana	Marine cyclopoids	Mixed ciliates		17	0.16	[28]
Oithona nana	Marine cyclopoids	Mixed ciliates		15	0.05	[28]
Oithona nana	Marine cyclopoids	Mixed ciliates		9		[51]
Oithona nana	Marine cyclopoids	Mixed ciliates		16		[51]
Oithona nana	Marine cyclopoids	Mixed ciliates		8		[51]
Oithona nana	Marine cyclopoids	Mixed ciliates		2		[51]
Oithona nana	Marine cyclopoids	Mixed ciliates		1		[51]
Oithona nana	Marine cyclopoids	Mixed ciliates		2		[51]
Oithona similis	Marine cyclopoids	*Myrionecta* spp. <30 µm		3	0.002	[18]
Oithona similis	Marine cyclopoids	*Myrionecta* spp. <30 µm		14	0.001	[18]
Oithona similis	Marine cyclopoids	*Myrionecta* spp. <30 µm		26	0.01	[18]
Oithona similis	Marine cyclopoids	*Myrionecta* spp. <30 µm		4	0.0005	[18]
Oithona similis	Marine cyclopoids	*Myrionecta* spp. <30 µm		24	0.005	[18]
Oithona similis	Marine cyclopoids	*Myrionecta* spp. >30 µm		12	0.02	[18]
Oithona similis	Marine cyclopoids	*Myrionecta* spp. >30 µm		27	0.07	[18]
Oithona similis	Marine cyclopoids	*Myrionecta* spp. >30 µm		21	0.02	[18]
Oithona similis	Marine cyclopoids	*Myrionecta* spp. >30 µm		19	0.02	[18]
Oithona similis	Marine cyclopoids	*Strombidium* spp. (<20 µm)		13	0.01	[18]
Oithona similis	Marine cyclopoids	*Strombidium* spp. (<20 µm)		2	0.002	[18]
Oithona similis	Marine cyclopoids	*Strombidium* spp. (<20 µm)		11	0.002	[18]
Oithona similis	Marine cyclopoids	*Strombidium* spp. (<20 µm)		17	0.003	[18]
Oithona similis	Marine cyclopoids	*Strombidium* spp. (<20 µm)		13	0.01	[18]
Oithona similis	Marine cyclopoids	*Strombidium* spp. (<20 µm)		15	0.01	[18]
Oithona similis	Marine cyclopoids	*Strombidium* spp. (<20 µm)		9	0.005	[18]
Oithona similis	Marine cyclopoids	*Strombidium* spp. (<20 µm)		4	0.001	[18]
Oithona similis	Marine cyclopoids	*Strombidium* spp. (<20 µm)		4	0.0007	[18]
Oithona similis	Marine cyclopoids	*Strombidium* spp. (<20 µm)		9	0.001	[18]
Oithona similis	Marine cyclopoids	*Strombidium* spp. (<20 µm)		6	0.0007	[18]
Oithona similis	Marine cyclopoids	*Strombidium* spp. (<20 µm)		4	0.0005	[18]
Oithona similis	Marine cyclopoids	*Strombidium* spp. (<20 µm)		14	0.001	[18]
Oithona similis	Marine cyclopoids	*Strombidium* spp. (20-30 µm)		3	0.011	[18]
Oithona similis	Marine cyclopoids	*Strombidium* spp. (20-30 µm)		14	0.049	[18]
Oithona similis	Marine cyclopoids	*Strombidium* spp. (20-30 µm)		5	0.014	[18]
Oithona similis	Marine cyclopoids	*Strombidium* spp. (20-30 µm)		13	0.053	[18]
Oithona similis	Marine cyclopoids	*Strombidium* spp. (20-30 µm)		14	0.038	[18]

Oithona similis	Marine cyclopoids	*Strombidium* spp. (20-30 µm)		22	0.057	[18]
Oithona similis	Marine cyclopoids	*Strombidium* spp. (20-30 µm)		12	0.044	[18]
Oithona similis	Marine cyclopoids	*Strombidium* spp. (20-30 µm)		13	0.022	[18]
Oithona similis	Marine cyclopoids	*Strombidium* spp. (20-30 µm)		35	0.028	[18]
Oithona similis	Marine cyclopoids	*Strombidium* spp. (20-30 µm)		6	0.003	[18]
Oithona similis	Marine cyclopoids	*Strombidium* spp. (20-30 µm)		16	0.019	[18]
Oithona similis	Marine cyclopoids	*Strombidium* spp. (20-30 µm)		9	0.015	[18]
Oithona similis	Marine cyclopoids	*Strombidium* spp. (20-30 µm)		10	0.002	[18]
Oithona similis	Marine cyclopoids	*Strombidium* spp. (20-30 µm)		3	0.011	[18]
Oithona similis	Marine cyclopoids	Strombidium spp. (>30 µm)		21	0.040	[18]
Oithona similis	Marine cyclopoids	Mixed ciliates (>20 µm)		4	0.03	[52]
Oithona similis	Marine cyclopoids	Mixed ciliates (>20 µm)		5	0.05	[52]
Oithona similis	Marine cyclopoids	Mixed ciliates		3	0.053	[18]
Oithona similis	Marine cyclopoids	Mixed ciliates		16	0.129	[18]
Oithona similis	Marine cyclopoids	Mixed ciliates		9	0.078	[18]
Oithona similis	Marine cyclopoids	Mixed ciliates		12	0.066	[18]
Oithona similis	Marine cyclopoids	Mixed ciliates		8	0.062	[18]
Oithona similis	Marine cyclopoids	Mixed ciliates		15	0.091	[18]
Oithona similis	Marine cyclopoids	Mixed ciliates		4	0.026	[18]
Oithona similis	Marine cyclopoids	Mixed ciliates		8	0.008	[18]
Oithona similis	Marine cyclopoids	Mixed ciliates		19	0.035	[18]
Oithona similis	Marine cyclopoids	Mixed ciliates		5	0.009	[18]
Oithona similis	Marine cyclopoids	Mixed ciliates		6	0.012	[18]
Oithona similis	Marine cyclopoids	Mixed ciliates		7	0.018	[18]
Oithona similis	Marine cyclopoids	Mixed ciliates		0	0	[18]
Oithona similis	Marine cyclopoids	Mixed ciliates		2	0.001	[18]
Oithona similis	Marine cyclopoids	Mixed ciliates		24	0.017	[18]
Oithona similis	Marine cyclopoids	Mixed ciliates		3	0.002	[18]
Oithona similis	Marine cyclopoids	Mixed ciliates		19	0.009	[18]
Oithona similis	Marine cyclopoids	Mixed ciliates		2	0.001	[18]
Oithona similis	Marine cyclopoids	Mixed ciliates		6	0.003	[18]
Oithona similis	Marine cyclopoids	Mixed ciliates		4	0.25	[18]
Oithona similis	Marine cyclopoids	Mixed ciliates		4	0.3	[18]
Oithona similis	Marine cyclopoids	Mixed ciliates		121	0.11	[18]
Oithona similis	Marine cyclopoids	Mixed ciliates		75	0.03	[18]
Oithona similis	Marine cyclopoids	Mixed ciliates		4	0.03	[18]
Oithona spp	Marine cyclopoids	Mixed ciliates		5	0.05	[18]
Oithona spp	Marine cyclopoids	Mixed ciliates		7		[18]
Oithona spp	Marine cyclopoids	Mixed ciliates		19	0.12	[18]
Oithona spp	Marine cyclopoids	Mixed ciliates		12	0.01	[18]
Oithona spp	Marine cyclopoids	Mixed ciliates		7	0.04	[18]
Oithona spp.	Marine cyclopoids	Aloricate ciliates	150	354		[53]
Oithona spp.	Marine cyclopoids	Aloricate ciliates	150	37		[53]
Oithona spp.	Marine cyclopoids	Aloricate ciliates	150	156		[53]
Oithona spp.	Marine cyclopoids	Aloricate ciliates	150	33		[53]
Oithona spp.	Marine cyclopoids	Aloricate ciliates	150	144		[53]
Oithona spp.	Marine cyclopoids	Tintinnids	225	239		[53]
Oithona spp.	Marine cyclopoids	Tintinnids	225	4		[53]
Oithona spp.	Marine cyclopoids	Tintinnids	225	91		[53]
Oithona spp.	Marine cyclopoids	Tintinnids	225	73		[53]

Data sources

1 Hansen, P. J., Bjørnsen, P. K. & Hansen, B. W. Zooplankton grazing and growth: Scaling within the 2-2,000-µm body size range. *Limnol. Oceanogr.* **42**, 687-704, doi:10.4319/lo.1997.42.4.0687 (1997).

2 Jack, J. D. & Gilbert, J. J. Susceptibilities of different-sized ciliates to direct suppression by small and large cladocerans. *Freshwater Biol.* **29**, 19-29 (1993).

3 Burns, C. W. & Schallenberg, M. Impacts of nutrients and zooplankton on the microbial food web of an ultra-oligotrophic lake. *J. Plankton Res.* **20**, 1501-1525, doi:https://doi.org/10.1093/plankt/20.8.1501 (1998).

4 Burns, C. W. & Schallenberg, M. Calanoid copepods versus cladocerans: Consumer effects on protozoa in lakes of different trophic status. *Limnol. Oceanogr.* **46**, 1558-1565, doi:https://doi.org/10.4319/lo.2001.46.6.1558 (2001).

5 Burns, C. W. & Schallenberg, M. Relative impacts of copepods, cladocerans and nutrients on the microbial food web of a mesotrophic lake. *J. Plankton Res.* **18**, 683-714, doi:https://doi.org/10.1093/plankt/18.5.683 (1996).

6 Wickham, S. A. The direct and indirect impact of *Daphnia* and cyclops on a freshwater microbial food web. *J. Plankton Res.* **20**, 739-755 (1998).

7 Porter, K. G., Pace, M. L. & Battey, F. J. Ciliate protozoans as links in freshwater planktonic food chains. *Nature* **277**, 563-565 (1979).

8 McMahon, J. & Rigler, F. Feeding rate of *Daphnia magna* straus in different foods labeled with radioactive phosphorus. *Limnol. Oceanogr.* **10**, 105-113 (1965).

9 Wiackowski, K., Brett, M. T. & Goldman, C. R. Differential effects of zooplankton species on ciliate community structure. *Limnol. Oceanogr.* **39**, 486-492 (1994).

10 Lu, X. & Weisse, T. Top-down control of planktonic ciliates by microcrustacean predators is stronger in lakes than in the ocean. *Scientific Reports* (this study) (2022).

11 Adrian, R. & Schneider-Olt, B. Top-down effects of crustacean zooplankton on pelagic microorganisms in a mesotrophic lake. *J. Plankton Res.* **21**, 2175-2190, doi:https://doi.org/10.1093/plankt/21.11.2175 (1999).

12 Hartmann, H. J., Taleb, H., Aleya, L. & Lair, N. Predation on ciliates by the suspension-feeding calanoid copepod *Acanthodiaptomus denticornis*. *Can. J. Fish. Aquat. Sci.* **50**, 1382-1393 (1993).

13 Burns, C. W. & Gilbert, J. J. Predation on ciliates by freshwater calanoid copepods: rates of predation and relative vulnerabilities of prey. *Freshwater Biol.* **30**, 377-393, doi:https://doi.org/10.1111/j.1365-2427.1993.tb00822.x (1993).

14 Jack, J. D. & Gilbert, J. J. Effects of metazoan predators on ciliates in freshwater plankton communities. *J. Euk. Microbiol.* **44**, 194-199 (1997).

15 Wickham, S. A. *Cyclops* predation on ciliates: species-specific differences and functional responses. *J. Plankton Res.* **17**, 1633-1646 (1995).

16 Kumar, R. Effects of *Mesocyclops thermocyclopoides* (Copepoda: Cyclopoida) predation on the population growth patterns of different prey species. *J. Freshw. Ecol.* **18**, 383-393, doi:10.1080/02705060.2003.9663974 (2003).

17 Broglio, E., Saiz, E., Calbet, A., Trepat, I. & Alcaraz, M. Trophic impact and prey selection by crustacean zooplankton on the microbial communities of an oligotrophic coastal area (NW Mediterranean Sea). *Aquat. Mic. Ecol.* **35**, 65-78 (2004).

18 Castellani, C., Irigoien, X., Harris, R. P. & Lampitt, R. S. Feeding and egg production of *Oithona similis* in the North Atlantic. *Mar. Ecol. Prog. Ser.* **288**, 173-182 (2005).

19 Ayukai, T. Predation by *Acartia clausi* (Copepoda: Calanoida) on two species of tintinnids. *Mar. Microb. Food Webs* **2**, 45-52 (1987).

20 Gismervik, I. Top-down impact by copepods on ciliate numbers and persistence depends on copepod and ciliate species composition *J. Plankton Res.* **28**, 499-507 (2006).

21 Fileman, E., Petropavlovsky, A. & Harris, R. Grazing by the copepods *Calanus helgolandicus* and *Acartia claus*i on the protozooplankton community at station L4 in the Western English Channel. *J. Plankton Res.* **32**, 709-724, doi:https://doi.org/10.1093/plankt/fbp142 (2010).

22 Tiselius, P. Contribution of aloricate ciliates to the diet of *Acartia clausi* and *Centropages hamatus* in coastal waters. *Mar. Ecol. Prog. Ser.* **56**, 49-56 (1989).

23 Turner, J. T. & Anderson, D. M. Zooplankton grazing during dinoflagellate blooms in a Cape Cod embayment, with observations of oredation upon tintinnids by copepods. *Mar. Ecol.* **4**, 359-374 (1983).

24 Stoecker, D. K. & Egloff, D. A. Predation by *Acartia tonsa* Dana on planktonic ciliates and rotifers. *J. Exp. Mar. Biol. Ecol.* **110**, 53-68 (1987).

25 Robertson, J. R. Predation by estuarine zooplankton on tintinnid ciliates. *Estuar. Coast. Shelf Sci.* **16**, 27-36 (1983).

26 Stoecker, D. K. & Sanders, N. K. Differential grazing by *Acartia tonsa* on a dinoflagellate and a tintinnid. *J. Plankton Res.* **7**, 85-100 (1985).

27 Jonsson, P. R. & Tiselius, P. Feeding behaviour, prey detection and capture efficiency of the copepod *Acartia tonsa* feeding on planktonic ciliates. *Mar. Ecol. Prog. Ser.* **60**, 35-44 (1990).

28 Brun, P. G., Payne, M. R. & Kiørboe, T. A trait database for marine copepods. *Earth System Science Data* **9**, 99-113, doi:https://doi.org/10.5194/essd-9-99-2017 (2017).

29 Vargas, C. A. & González, H. E. Plankton community structure and carbon cycling in a coastal upwelling system. I. Bacteria, microprotozoans and phytoplankton in the diet of copepods and appendicularians. *Aquat. Mic. Ecol.* **34**, 151-164 (2004).

30 Bollens, G. C. R. & Penry, D. L. Feeding dynamics of *Acartia* spp. copepods in a large, temperate estuary (San Francisco Bay, CA). *Mar. Ecol. Prog. Ser.* **257**, 139-158, doi:https://doi.org/10.3354/meps257139 (2003).

31 Lonsdale, D. J. *et al.* Food web interactions in the plankton of Long Island bays, with preliminary observations on brown tide effects. *Mar. Ecol. Prog. Ser.* **134**, 247-263, doi:https://doi.org/10.3354/meps134247 (1996).

32 Batten, S. D., Fileman, E. S. & Halvorsen, E. The contribution of microzooplankton to the diet of mesozooplankton in an upwelling filament off the north west coast of Spain. *Progr. Oceanogr.* **51**, 385-398 (2001).

33 Atkinson, A. Subantarctic copepods in an oceanic, low chlorophyll environment: ciliate predation, food selectivity and impact on prey populations. *Mar. Ecol. Prog. Ser.* **130**, 85-96 (1996).

34 Levinsen, H., Turner, J. T., Nielsen, T. G. & Hansen, B. W. On the trophic coupling between protists and copepods in arctic marine ecosystems. *Mar. Ecol. Prog. Ser.* **204**, 65-77, doi:https://doi.org/10.3354/meps204065 (2000).

35 Ohman, M. D. & Runge, J. A. Sustained fecundity when phytoplankton resources are in short supply: omnivory by *Calanus finmarchicus* in the Gulf of St. Lawrence. *Limnol. Oceanogr.* **39**, 21-36, doi:https://doi.org/10.4319/lo.1994.39.1.0021 (1994).

36 Nejstgaard, J. C., Gismervik, I. & Solberg, P. T. Feeding and reproduction by *Calanus finmarchicus*, and microzooplankton grazing during mesocosm blooms of diatoms and the coccolithophore *Emiliania huxleyi*. *Mar. Ecol. Prog. Ser.* **147**, 197-217, doi:https://doi.org/10.3354/meps147197 (1997).

37 Vincent, D. & Hartmann, H. J. Contribution of ciliated microprotozoans and dinoflagellates to the diet of three copepod species in the Bay of Biscay. *Hydrobiologia* **443**, 193-204, doi:https://doi.org/10.1023/A:1017502813154 (2001).

38 Fessenden, L. & Cowles, T. J. Copepod predation on phagotrophic ciliates in Oregon coastal waters. *M Mar. Ecol. Prog. Ser.* **107**, 103-103 (1994).

39 Turner, J. T. & Granéli, E. Zooplankton feeding ecology: grazing during enclosure studies of phytoplankton blooms from the west coast of Sweden. *J. Exp. Mar. Biol. Ecol.* **157**, 19-31, doi:https://doi.org/10.1016/0022-0981(92)90071-H (1992).

40 Wiadnyana, N. N. & Rassoulzadegan, F. Selective feeding of *Acartia clausi* and *Centropages typicus* on microzooplankton. *Mar. Ecol. Prog. Ser.* **53**, 37-45 (1989).

41 Verity, P. & Paffenhofer, G.-A. On assessment of prey ingestion by copepods. *J. Plankton Res.* **18**, 1767-1779, doi:https://doi.org/10.1093/plankt/18.10.1767 (1996).

42 Berk, S., Brownlee, D., Heinle, D., Kling, H. & Colwell, R. Ciliates as a food source for marine planktonic copepods. *Microb. Ecol.* **4**, 27-40 (1977).

43 Liu, H., Dagg, M. J. & Strom, S. Grazing by the calanoid copepod *Neocalanus cristatus* on the microbial food web in the coastal Gulf of Alaska. *J. Plankton Res.* **27**, 647-662, doi:https://doi.org/10.1093/plankt/fbi039 (2005).

44 Gifford, D. J. & Dagg, M. J. The microzooplankton-mesozooplankton link: consumption of planktonic protozoa by the calanoid copepods *Acartia tonsa* Dana and *Neocalanus plumchrus* Murukawa. *Mar. Microb. Food Webs* **5**, 161-177 (1991).

45 Pérez, M., Dolan, J. & Fukai, E. Planktonic oligotrich ciliates in the NW Mediterranean: growth rates and consumption by copepods. *Mar. Ecol. Prog. Ser.* **155**, 89-101 (1997).

46 Zeldis, J., James, M. R., Grieve, J. & Richards, L. Omnivory by copepods in the New Zealand Subtropical Frontal Zone. *J. Plankton Res.* **24**, 9-23, doi:https://doi.org/10.1093/plankt/24.1.9 (2002).

47 Dagg, M., Strom, S. & Liu, H. High feeding rates on large particles by *Neocalanus flemingeri* and *N. plumchrus*, and consequences for phytoplankton community structure in the subarctic Pacific Ocean. *Deep Sea Res. Part I Oceanogr. Res. Pap.* **56**, 716-726, doi:https://doi.org/10.1016/j.dsr.2008.12.012 (2009).

48 Suzuki, K., Nakamura, Y. & Hiromi, J. Feeding by the small calanoid copepod *Paracalanus* sp. on heterotrophic dinoflagellates and ciliates. *Aquat. Microb. Ecol.* **17**, 99-103 (1999).

49 Hansen, B., Christiansen, S. & Pedersen, G. Plankton dynamics in the marginal ice zone of the central Barents Sea during spring: carbon flow and structure of the grazer food chain. *Polar Biol.* **16**, 115-128, doi:https://doi.org/10.1007/BF02390432 (1996).

50 Saiz, E., Griffell, K., Calbet, A. & Isari, S. Feeding rates and prey : predator size ratios of the nauplii and adult females of the marine cyclopoid copepod O*ithona davisae*. *Limnol. Oceanogr.* **59**, 2077-2088, doi:https://doi.org/10.4319/lo.2014.59.6.2077 (2014).

51 Atienza, D., Saiz, E. & Calbet, A. Feeding ecology of the marine cladoceran *Penilia avirostris:* natural diet, prey selectivity and daily ration. *Mar. Ecol. Prog. Ser.* **315**, 211-220 (2006).

52 Nakamura, Y. & Turner, J. T. Predation and respiration by the small cyclopoid copepod *Oithona similis*: How important is feeding on ciliates and heterotrophic flagellates? *J. Plankton Res.* **19**, 1275-1288, doi:https://doi.org/10.1093/plankt/19.9.1275 (1997).

53 Lonsdale, D. J., Caron, D. A., Dennett, M. R. & Schaffner, R. Predation by *Oithona* spp. on protozooplankton in the Ross Sea, Antarctica. *Deep Sea Research Part II: Topical Studies in Oceanography* **47**, 3273-3283, doi:https://doi.org/10.1016/S0967-0645(00)00068-0 (2000).

54 Kunzmann, A. J., Ehret, H., Yohannes, E., Straile, D. & Rothhaupt, K.-O. Calanoid copepod grazing affects plankton size structure and composition in a deep, large lake. *J. Plankton Res.* **41**, 955-966, doi: https://doi.org/10.1093/plankt/fbz067 (2019).

Supplementary Table S1. Linear model results for log_{10}–transformed ingestion rates (all data without outliers, n=72) and clearance rates of the three microcrustacean predators (all data without outliers, n=72). Significant effects in bold face.

**Ingestion rates**			
**Predator**	**R^2**	**p-value (Ciliates)**	**p-value (Predator)**
Daphnia	**0.893**	**<0.001**	-
Eudiaptomus	**0.767**	**<0.001**	-
Cyclops	**0.950**	**<0.001**	-
All predators	**0.870**	**<0.001**	0.150
**Clearance rates**			
**Predator**	**R^2**	**p-value (Ciliates)**	**p-value (Predator)**
Daphnia	0.276	0.150	-
Eudiaptomus	0.267	0.101	-
Cyclops	0.073	0.826	-
All predators	**0.269**	**0.020**	0.490

Supplementary Table S2. Model fits of power curves ($y = ax^b$), exponential curves ($y = ae^{bx}$) and ordinary least-squares regressions ($y = kx + c$) for $\log_{10}$–transformed ingestion and clearance rates rates of the three microcrustacean predators in relation to log-ciliate size. Significant parameter estimates (± standard error, SE) are in bold face; adj. R^2 = adjusted R^2.

Predator	Model	AIC score	R²	residual SE	a	b	k	c	p (a)	p (b)	p (k)	p (c)
**Ingestion rates**												
	**Power curve**	21.8	0.832	0.372	0.126 ± 0.068	**4.750** ± 0.912			0.08	**< 0.001**		
Daphnia	**Exponential curve**	19.8	0.849	0.353	0.009 ± 0.009	**2.991** ± 0.589			0.30	**< 0.001**		
	**Linear regression**	27.2	**0.777**	0.428			**2.919** ± 0.379	**-3.183** ± 0.626			**< 0.001**	**< 0.001**
	**Power curve**	29.6	0.750	0.371	**0.329** ± 0.091	**3.252** ± 0.485			**0.01**	**< 0.001**		
Eudiaptomus	**Exponential curve**	30.7	0.750	0.378	0.059 ± 0.032	**2.018** ± 0.306			0.08	**< 0.001**		
	**Linear regression**	28.9	**0.765**	0.366			**2.970** ± 0.317	**-3.130** ± 0.526			**< 0.001**	**< 0.001**
	**Power curve**	18.3	**0.852**	0.318	**0.274** ± 0.071	**3.652** ± 0.462			**< 0.001**	**< 0.001**		
Cyclops	**Exponential curve**	20.5	0.838	0.334	0.040 ± 0.020	**2.267** ± 0.289			0.061	**< 0.001**		
	**Linear regression**	14.1	**0.876**	0.292			**3.380** ± 0.272	**-3.764** ± 0.441			**< 0.001**	**< 0.001**
	**Power curve**	62.2	**0.796**	0.362	**0.273** ± 0.049	**3.562** ± 0.316			**< 0.001**	**< 0.001**		
All predators	**Exponential curve**	64.4	**0.790**	0.367	**0.040** ± 0.014	**2.225** ± 0.193			**< 0.05**	**< 0.001**		
	**Linear regression**	61.6	**0.798**	0.360			**3.074** ± 0.185	**-3.327** ± 0.305			**< 0.001**	**< 0.001**
**Clearance rates**												
Daphnia	**Power curve**	14.9	**0.148**	0.294	**1.691** ± 0.237	**-0.600** ± 0.296			**< 0.001**	**0.05**		
	**Exponential curve**	15.1	0.140	0.296	**2.376** ± 0.754	-0-384			**< 0.01**	0.06		
	**Linear regression**	15.3	0.090	0.297			-0.483 ± 0.263	**2.062** ± 0.432			0.08	**< 0.001**
Eudiaptomus	**Power curve**	29.1	0.252	0.368	**2.050** ± 0.332	**-1.118** ± 0.348			**< 0.001**	**< 0.001**		
	**Exponential curve**	29.1	0.254	0.367	**4.036** ± 1.483	**-0.744** ± 0.232			**0.011**	**< 0.01**		
	**Linear regression**	29.0	**0.257**	0.367			**-0.969** ± 0.317	**2.797** ± 0.527			**< 0.01**	**< 0.001**
Cyclops	**Power curve**	**-6.45**	0.019	0.190	**1.407** ± 0.135	-0.130 ± 0.199			**< 0.001**	0.520		
	**Exponential curve**	**-6.45**	0.019	0.190	**1.524** ± 0.324	-0.087 ± 0.132			**< 0.001**	0.516		
	**Linear regression**	**-6.46**	0.019	0.190			-0.116 ± 0.177	**1.511** ± 0.287			0.520	**< 0.001**
All predators	**Power curve**	40.0	**0.146**	0.304	**1.716** ± 0.142	**-0.650** ± 0.175			**< 0.001**	**< 0.001**		
	**Exponential curve**	40.1	**0.145**	0.304	**2.529** ± 0.471	**-0.429** ± 0.116			**< 0.001**	**< 0.001**		
	**Linear regression**	40.2	**0.142**	0.305			**-0.556** ± 0.157	**2.170** ± 0.257			**< 0.001**	**< 0.001**

Supplementary Table S3. Linear model results for log_{10}–transformed clearance rates and ingestion rates of the six functional groups. Significant effects in bold face.

**Clearance rates**	
**Functional group**	**p-value**
FW cladocerans	**<0.001**
FW calanoids	**<0.001**
FW cyclopoids	0.78
Marine cladocerans	**<0.001**
Marine calanoids	**<0.001**
Marine cyclopoids	**<0.001**
All functional groups	**<0.001**
Habitat	**<0.001**
**Ingestion rates**	
**Functional group**	**p-value**
FW cladocerans	0.39
FW calanoids	**0.001**
FW cyclopoids	0.26
Marine cladocerans	0.41
Marine calanoids	**<0.001**
Marine cyclopoids	**<0.001**
All functional group	**<0.001**
Habitat	**0.54**

Supplementary Table S4. Pairwise comparison (Tukey test) of ingestion and clearance rates of the six functional groups of predators. Significant differences in bold face.

Ingestion rates					
	FW cladocerans	FW cyclopoids	Marine calanoids	Marine cladocerans	Marine cyclopoids
FW calanoids	0.954	0.869	**0.004**	0.961	**<0.0001**
FW cladocerans		0.716	0.250	0.999	0.910
FW cyclopoids			0.868	0.666	**<0.0001**
Marine calanoids				0.108	**<0.0001**
Marine cladocerans					0.592
Clearance rates					
FW calanoids	**<0.01**	0.999	**<0.0001**	**0.023**	**<0.001**
FW cladocerans		0.140	**<0.0001**	0.894	0.999
FW cyclopoids			**<0.0001**	0.082	0.082
Marine calanoids				**<0.0001**	**<0.0001**
Marine cladocerans					0.826

Supplementary Table S5. Model fits of power curves ($y = ax^b$), exponential curves ($y = ae^{bx}$) and least-squares linear regressions ($y = kx + c$) for log_{10}–transformed clearance rates of the six functional groups of microcrustacean predators in relation to log_{10}–transformed ciliate size. Significant parameters (± standard error, SE) are in bold face; dna denotes that no model fit was possible.

Functional group	Model	AIC score	R^2	residual SE	a	b	k	c	p (a)	p (b)	p (k)	p (c)
FW cladocerans	Power curve	103.0			dna	dna						
	Exponential curve	103.0			dna	dna						
	Linear regression	14.2	**0.457**	0.278			**-0.975** ± 0.188	**2.669** ± 0.340			**< 0.001**	**< 0.001**
FW calanoids	Power curve	88.8	0.059	0.452	**0.984** ± 0.167	**0.582** ± 0.306			**< 0.001**	0.06		
	Exponential curve	89.9	0.049	0.454	**0.782** ± 0.244	0.314 ± 0.179			**< 0.01**	0.08		
	Linear regression	89.5	0.055	0.453			0.541 ± 0.410	0.468 ± 0.240			0.06	0.19
FW cyclopoids	Power curve	38.4	**0.146**	0.431	**0.882** ± 0.195	**0.785** ± 0.377			**< 0.001**	**< 0.05**		
	Exponential curve	39.0	0.123	0.436	**0.662** ± 0.250	**0.410** ± 0.210			**< 0.05**	0.06		
	Linear regression	38.6	0.143	0.432			**0.632** ± 0.298	0.261 ± 0.518			**<0.05**	0.62
All FW predators	Power curve	180.2			dna	dna						
	Exponential curve	180.2			dna	dna						
	Linear regression	180.2			dna	dna						
Marine calanoids	Power curve	136.7			dna	dna						
	Exponential curve	136.0			dna	dna						
	Linear regression	135.8			dna	dna						
Marine cyclopoids	Power curve	26.4			dna	dna						
	Exponential curve	26.6			dna	dna						
	Linear regression	26.4			dna	dna						
All marine predators	Power curve	155.4			dna	dna						
	Exponential curve	155.1			dna	dna						
	Linear regression	155.1			dna	dna						
